# Supplementary material for: Novel Uracil-Based Inhibitors of Acetylcholinesterase with Potency for Treating Memory Impairment in an Animal Model of Alzheimer’s Disease
Source: Molecules. 2022 Nov 14;27(22):7855. doi: 10.3390/molecules27227855 (PMC9694926; doi:10.3390/molecules27227855)
Supplement: Supplementary file 1 [file molecules-27-07855-s001.zip › molecules-2000168-supplementary.pdf]

The Supplementary Data

Novel potent uracil-based inhibitors of acetylcholinesterase for the treatment of memory impairment in animal model of Alzheimer’s disease

Vyacheslav E. Semenov [1,*], Irina V. Zueva [1], , Sofya V. Lushchekina [2], Eduard G. Suleimanov [1], Lilya M. Gubaidullina [1], Marina M. Schulaeva [1], Oksana A. Lenina [1], Konstantin A. Petrov [1,3,*]

[1] Arbuzov Institute of Organic and Physical Chemistry, FRC Kazan Scientific Center of RAS, Arbuzov str. 8, Kazan 420088, Russia

[2] Emanuel Institute of Biochemical Physics, Kosygina st. 4, Moscow, 119334, Russia

[3] Kazan Federal University, Kremlyovskaya str., 18, Kazan, 420008, Russi*a*

***** Correspondence: sve@iopc.ru (V.E.S.); kpetrov2005@mail.ru (K.A.P.)

Table of contents

Pages

NMR study of the compounds synthesized……………………………………………..S2
NMR experiments details……………………………………………………………...S2
MS experiments details………………………………………………………………..S2
Figure S1. 1D ^{1}H NMR spectrum of **2a** in $CDCl_3$ at T = 303 ………….…………………S4
Figure S2. 1D ^{13}C NMR spectrum of **2a** in $CDCl_3$ at T = 303……………………………S4
Figure S3. MALDI-TOF mass spectrum of **2a**…………………………………….....S5
Figure S4. MALDI-TOF mass spectrum of **2b**………………………………………S5
Figure S5. 1D ^{1}H NMR spectrum of **2b** in $CDCl_3$ at T = 303 ……………………………S6
Figure S6. 1D ^{13}C NMR spectrum of **2b** in $CDCl_3$ at T = 303……………………......S6
Figure S7. 1D ^{1}H NMR spectrum of **2c** in $CDCl_3$ at T = 303……………………………..S7
Figure S8. 1D ^{13}C NMR spectrum of **2c** in $CDCl_3$ at T = 303……………………………S7
Figure S9. MALDI-TOF mass spectrum of **2c**…………………………………….....S8
Figure S10. MALDI-TOF mass spectrum of **2d**……………………………………S8
Figure S11. 1D ^{1}H NMR spectrum of **2d** in $CDCl_3$ at T = 303……………………………S9
Figure S12. 1D ^{13}C NMR spectrum of **2d** in $CDCl_3$ at T = 303…………………………..S9
Figure S13. 1D ^{1}H NMR spectrum of **3a** in $CDCl_3$ at T = 303…………………………S10
Figure S14. 1D ^{13}C NMR spectrum of **3a** in $CDCl_3$ at T = 303..………………………..S10
Figure S15. MALDI-TOF mass spectrum of **3a**………………………………..……S11
Figure S16. MALDI-TOF mass spectrum of **3b**……………………………....……S11
Figure S17. 1D ^{1}H NMR spectrum of **3b** in $CDCl_3$ at T = 303………………….………S12
Figure S18. 1D ^{13}C NMR spectrum of **3b** in $CDCl_3$ at T = 303………………...…….S12
Figure S19. 1D ^{1}H NMR spectrum of **3c** in $CDCl_3$ at T = 303……………….…………S13

Figure S20. 1D ^{13}C NMR spectrum of **3c** in $CDCl_3$ at T = 303……………………….S13
Figure S21. MALDI-TOF mass spectrum of **3c**……………………………………..……S14
Figure S22. MALDI-TOF mass spectrum of **4a**……………………………………...…S14
Figure S23. 1D ^{1}H NMR spectrum of **4a** in $CDCl_3$ at T = 303………………………..S15
Figure S24. 1D ^{13}C NMR spectrum of **4a** in $CDCl_3$ at T = 303………………...……S15
Figure S25. 1D ^{1}H NMR spectrum of **4b** in $CDCl_3$ at T = 303………………………..S16
Figure S26. 1D ^{13}C NMR spectrum of **4b** in $CDCl_3$ at T = 303……………….....…….S16
Figure S27. MALDI-TOF mass spectrum of **4b**……………………………………...…S17
Figure S28. MALDI-TOF mass spectrum of **2e**……………………………………...…S17
Figure S29. 1D ^{1}H NMR spectrum of **2e** in DMSO-d_6 at T = 303….…………………S18
Figure S30. 1D ^{1}H NMR spectrum of **2e** in D_2O at T = 303.…………………...……S18
Figure S31. 1D ^{13}C NMR spectrum of **2e** in D_2O at T = 303……………………...……S19
Figure S32. 1D ^{1}H NMR spectrum of **3d** in D_2O at T = 303……….…………………S19
Figure S32. 1D ^{1}H NMR spectrum of **3d** in DMSO-d_6 at T = 303….…………………S20
Figure S34. 1D ^{13}C NMR spectrum of **3d** in D_2O at T = 303...…………………...….S20
Figure S35. MALDI-TOF mass spectrum of **3d**……………………………………...…S21
Figure S36. MALDI-TOF mass spectrum of **2f**……………………………………...….S21
Figure S37. 1D ^{1}H NMR spectrum of **2f** in DMSO-d_6 at T = 303….…………………S22
Figure S38. 1D ^{13}C NMR spectrum of **2f** in DMSO-d_6 at T = 303….………………...S22
Figure S39. 1D ^{1}H NMR spectrum of **2f** in D_2O at T = 303…...…………………….S23
Figure S40. 1D ^{13}C NMR spectrum of **2f** in D_2O at T = 303……….………………....S23
NMR study of the stability of compound 2c………...S24

NMR and MS study of the compounds synthesized

NMR experiments details

All NMR experiments were performed with 400.1 MHz and 600.1 MHz for ^{1}H NMR, and 100.6 and 150.6 MHz for ^{13}C NMR spectrometer equipped with 5 mm diameter gradient inverse broad band probehead and a pulsed gradient unit capable of producing magnetic field pulse gradients in the *z*-direction of 53.5 $G \cdot cm^{-1}$. NMR experiments were carried out at 303 K. Chemical shifts (δ in ppm) were referenced to the solvents DMSO-d_6 (δ = 2.50 ppm for ^{1}H and 40.0 ppm for ^{13}C NMR), $CDCl_3$ (δ = 7.26 ppm for ^{1}H and 77.0 ppm for ^{13}C NMR) and D_2O (δ = 4.70 ppm for ^{1}H).

MS experiments details

MALDI-TOF mass spectra were recorded in a positive and negative ion mode on a Bruker ULTRAFLEX III mass spectrometer (Bruker Daltonic GmbH, Bremen, Germany) using *p*-nitroaniline as a matrix for 10^{-3} mg/ml solutions in MeOH. A Nd:YAG laser (λ = 355 nm, repetition rate 100 Hz) was used. The mass spectrum was obtained with an

accelerating voltage of 25 kV and an ion extraction delay time of 30 ns. The resulting mass spectrum was formed due to multiple laser irradiation of the crystal (50 shots). The metal target MTP AnchorChipTM was used. Portions (0.5 μl) of a 1% matrix solution in acetonitrile and sample solution were consecutively applied onto the target and evaporated. The polyethylene glycol was used to calibrate the mass scale of the device. The data was obtained using the FlexControl program and processed using the FlexAnalysis 3.0 program (Bruker Daltonik GmbH, Germany).

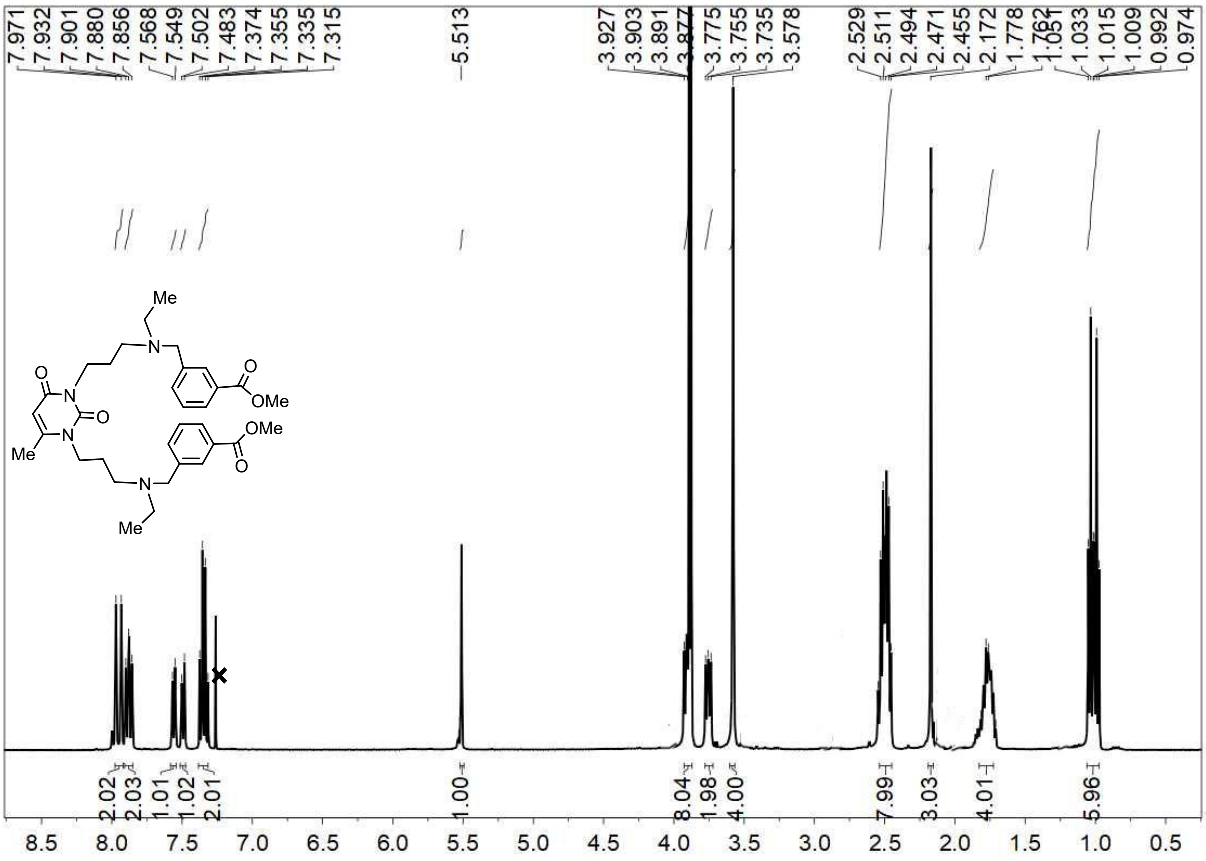

Figure S1. 1D ^{1}H NMR spectrum of **2a** in $CDCl_3$ (400 MHz) at T = 303 K. x - residual solvent peak.

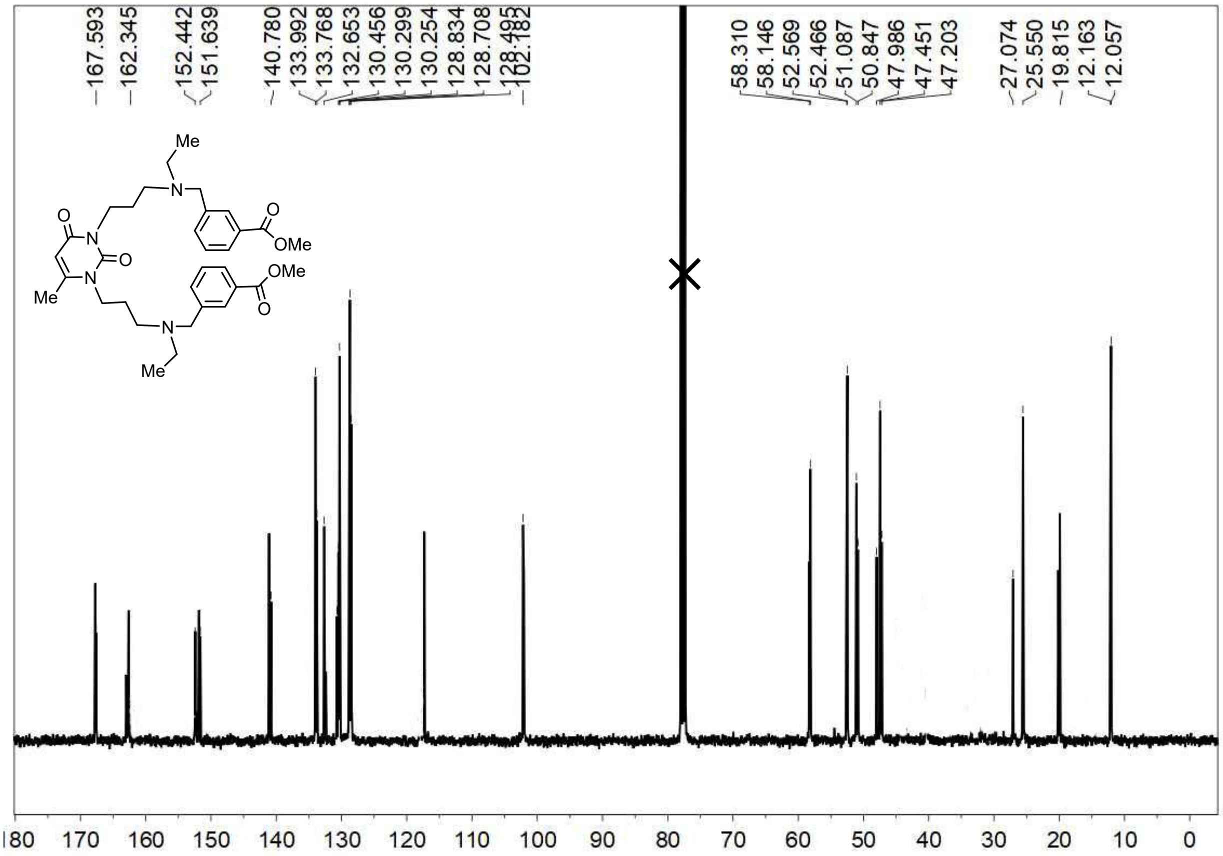

Figure S2. 1D ^{13}C NMR spectrum of **2a** in $CDCl_3$ at T = 303. x - residual solvent peak.

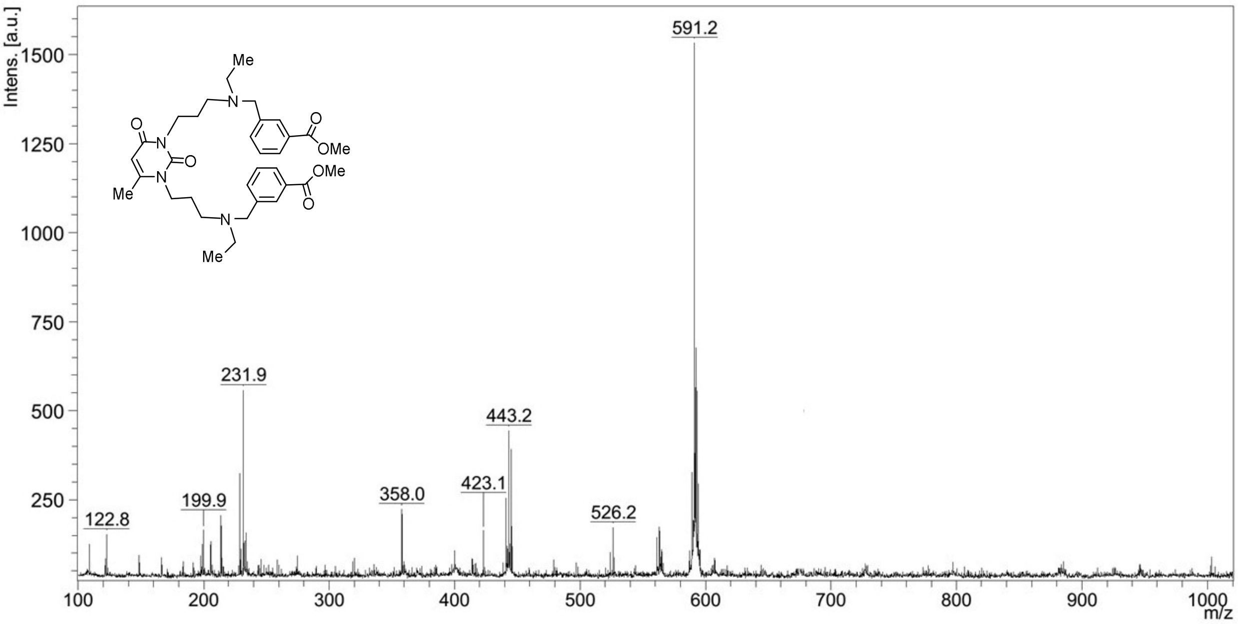

Figure S3. MALDI-TOF mass spectrum of **2a**.

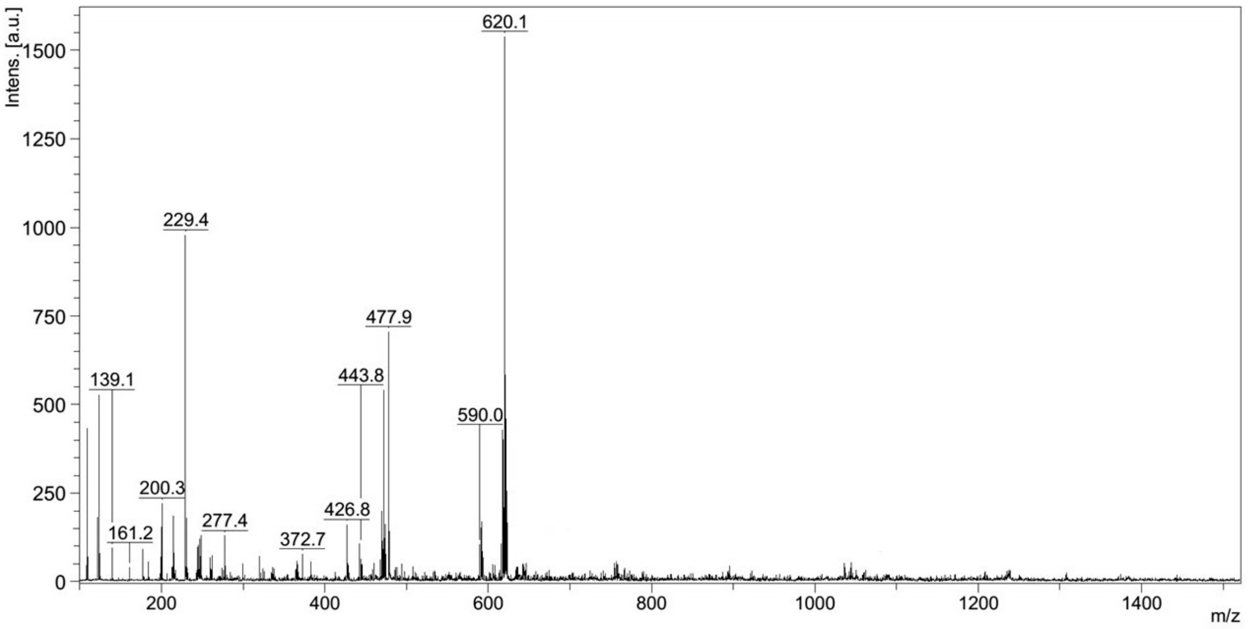

Figure S4. MALDI-TOF mass spectrum of **2b**.

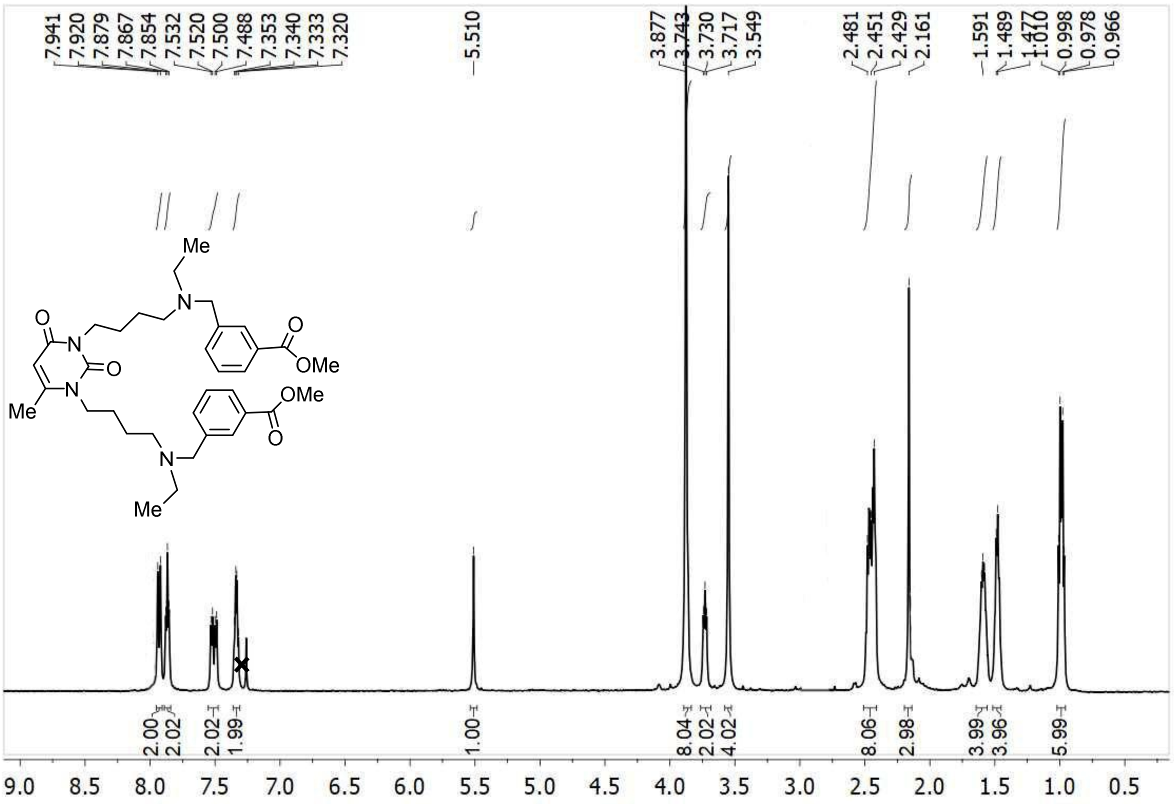

Figure S5. 1D ^{1}H NMR spectrum of **2b** in $CDCl_3$ (600 MHz) at T = 303 K. x - residual solvent peak.

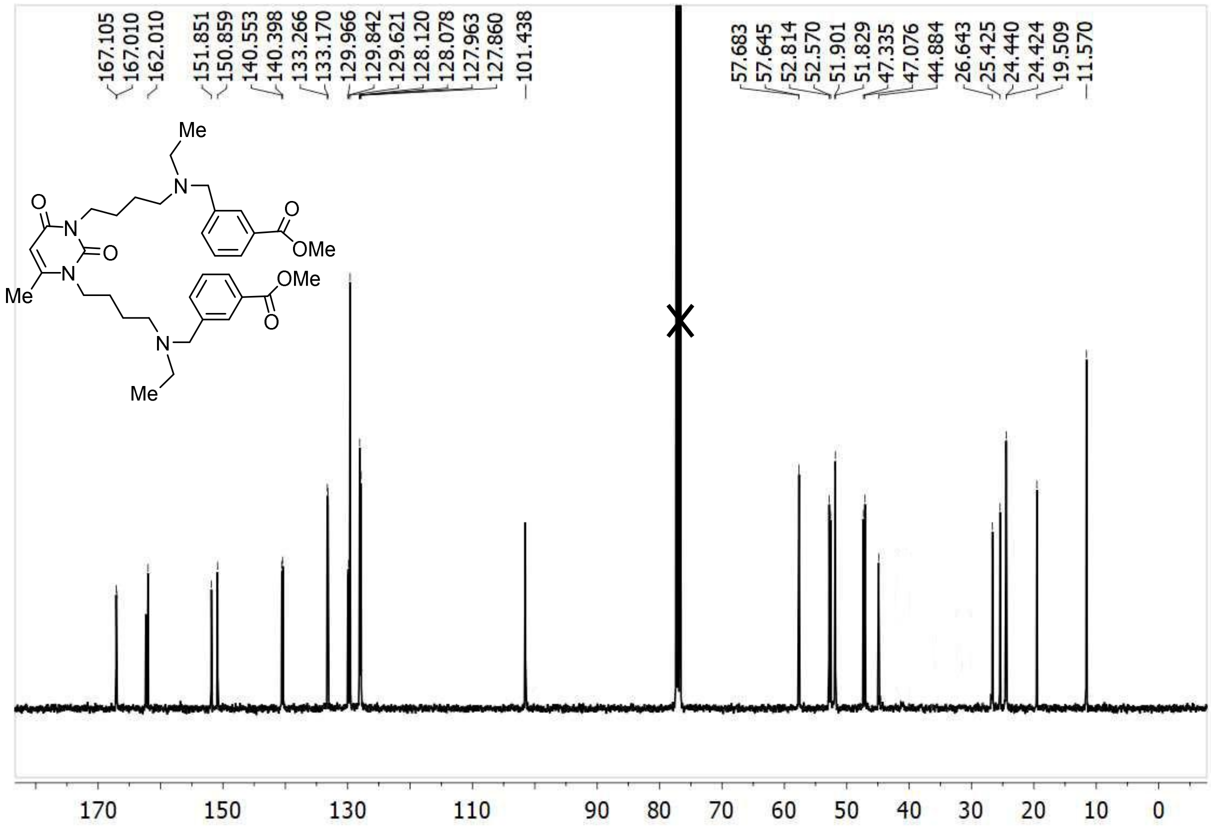

Figure S6. 1D ^{13}C NMR spectrum of **2b** in $CDCl_3$ (150 MHz) at T = 303. x - residual solvent peak.

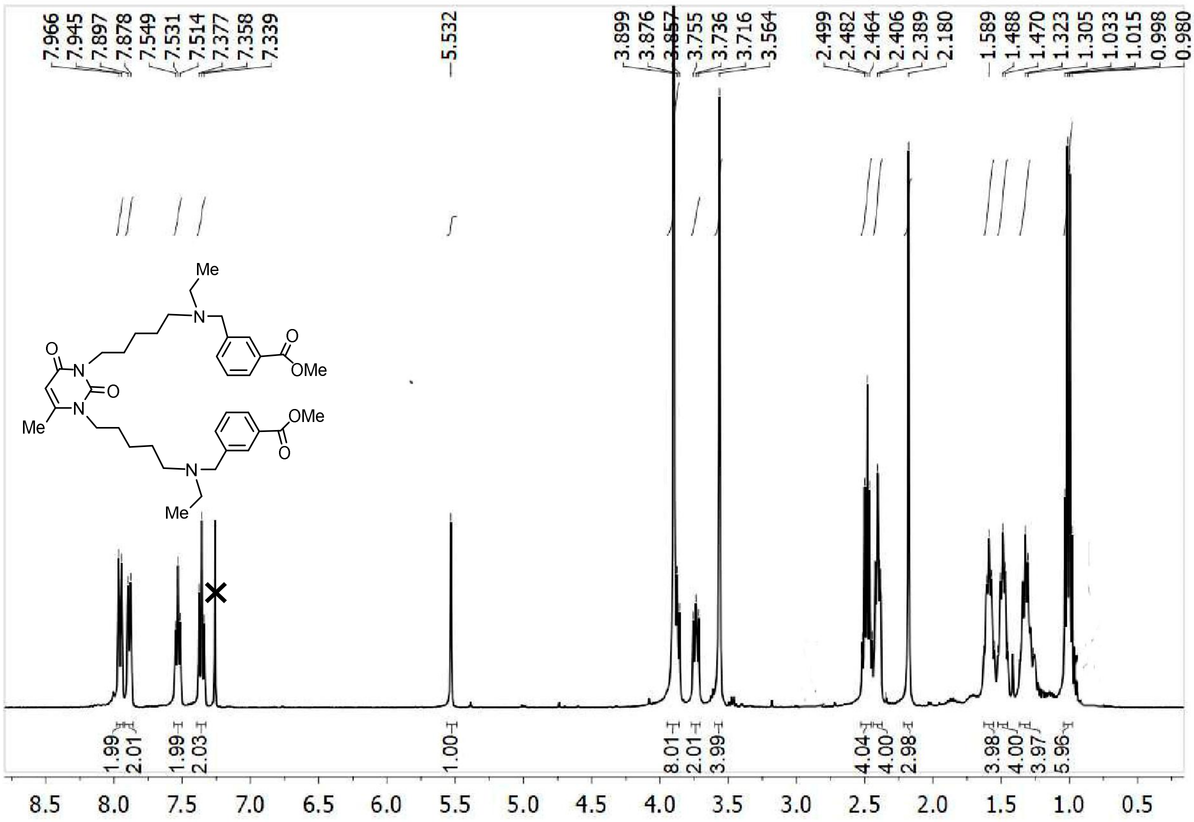

Figure S6. 1D ^{1}H NMR spectrum of **2c** in $CDCl_3$ (600 MHz) at T = 303 K. x - residual solvent peak.

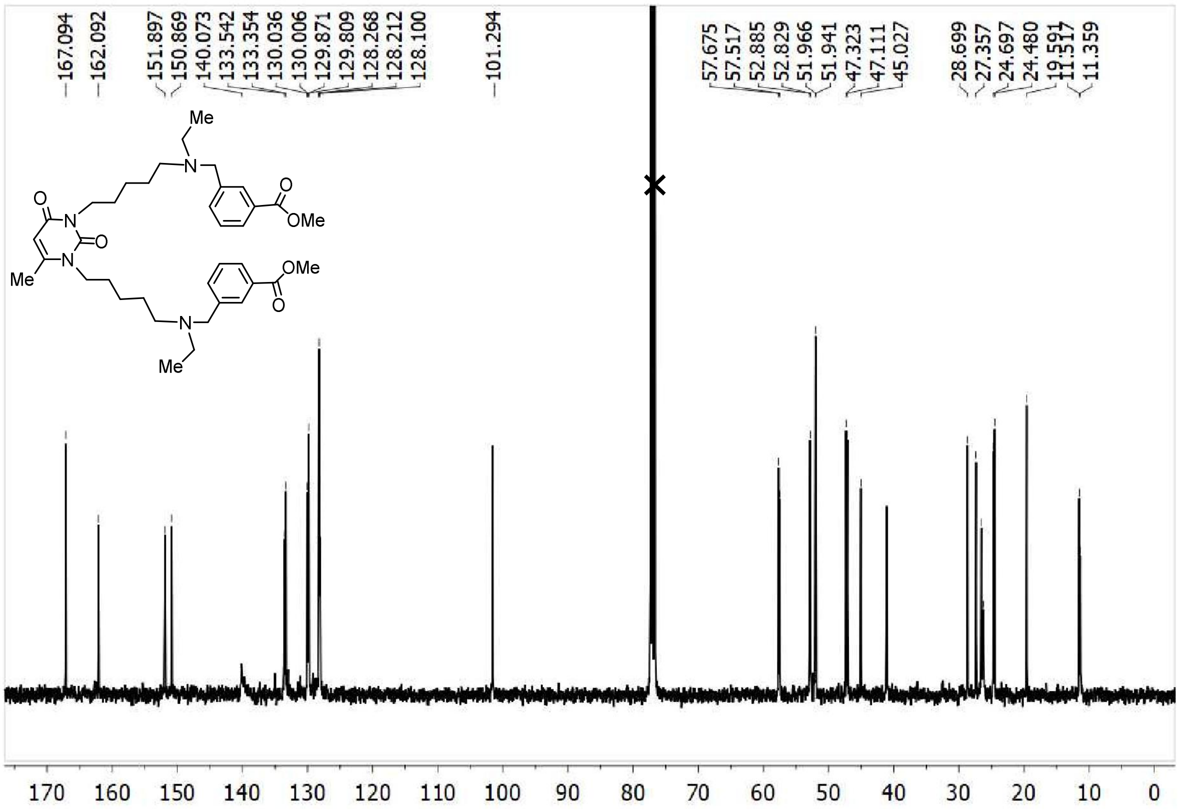

Figure S8. 1D ^{13}C NMR spectrum of **2c** in $CDCl_3$ (150 MHz) at T = 303. x - residual solvent peak.

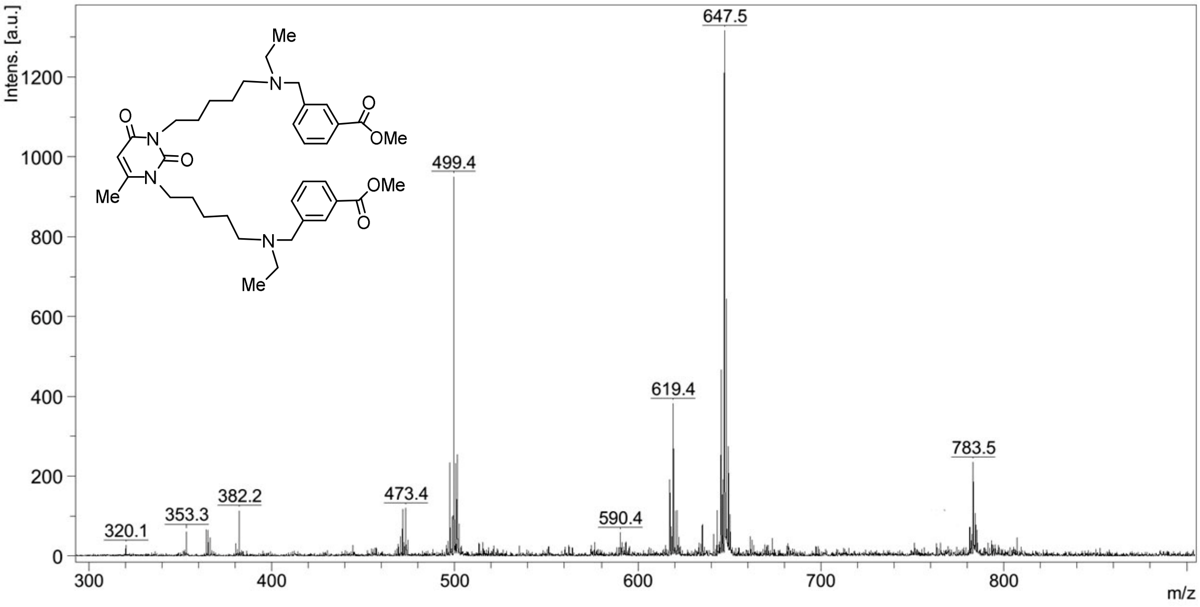

Figure S9. MALDI-TOF mass spectrum of **2c**.

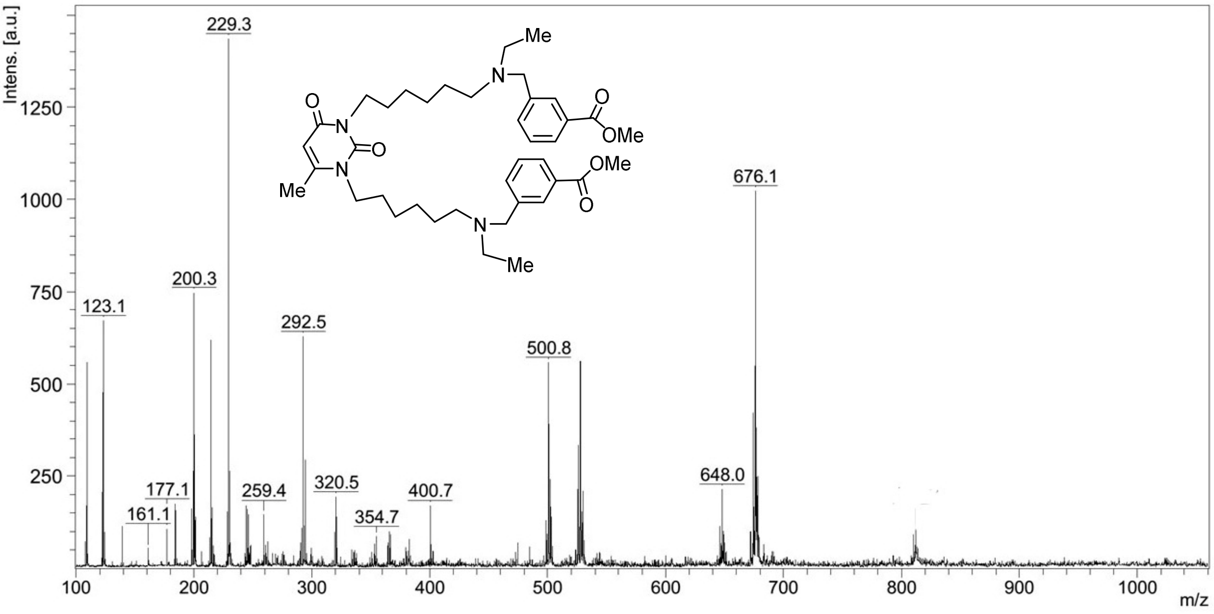

Figure S10. MALDI-TOF mass spectrum of **2d**.

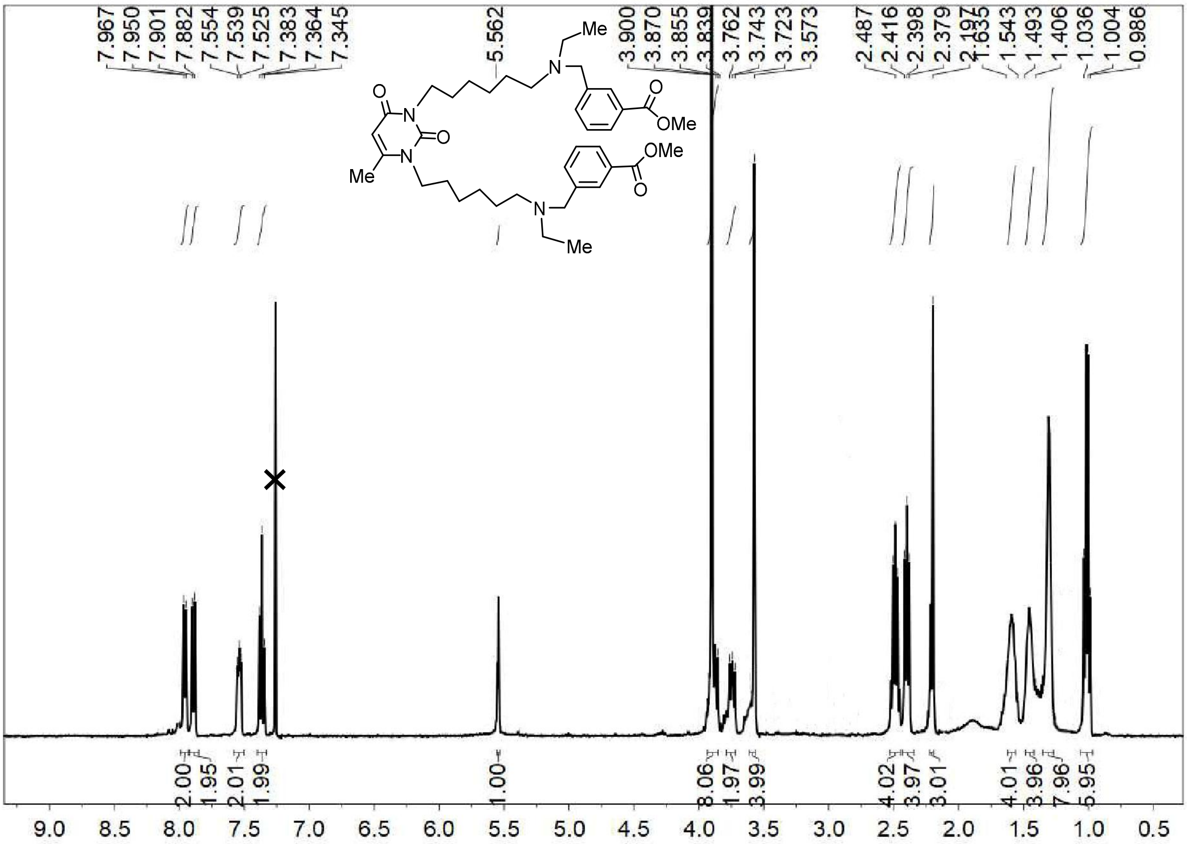

Figure S11. 1D ^{1}H NMR spectrum of **2d** in $CDCl_3$ (400 MHz) at T = 303 K. x - residual solvent peak.

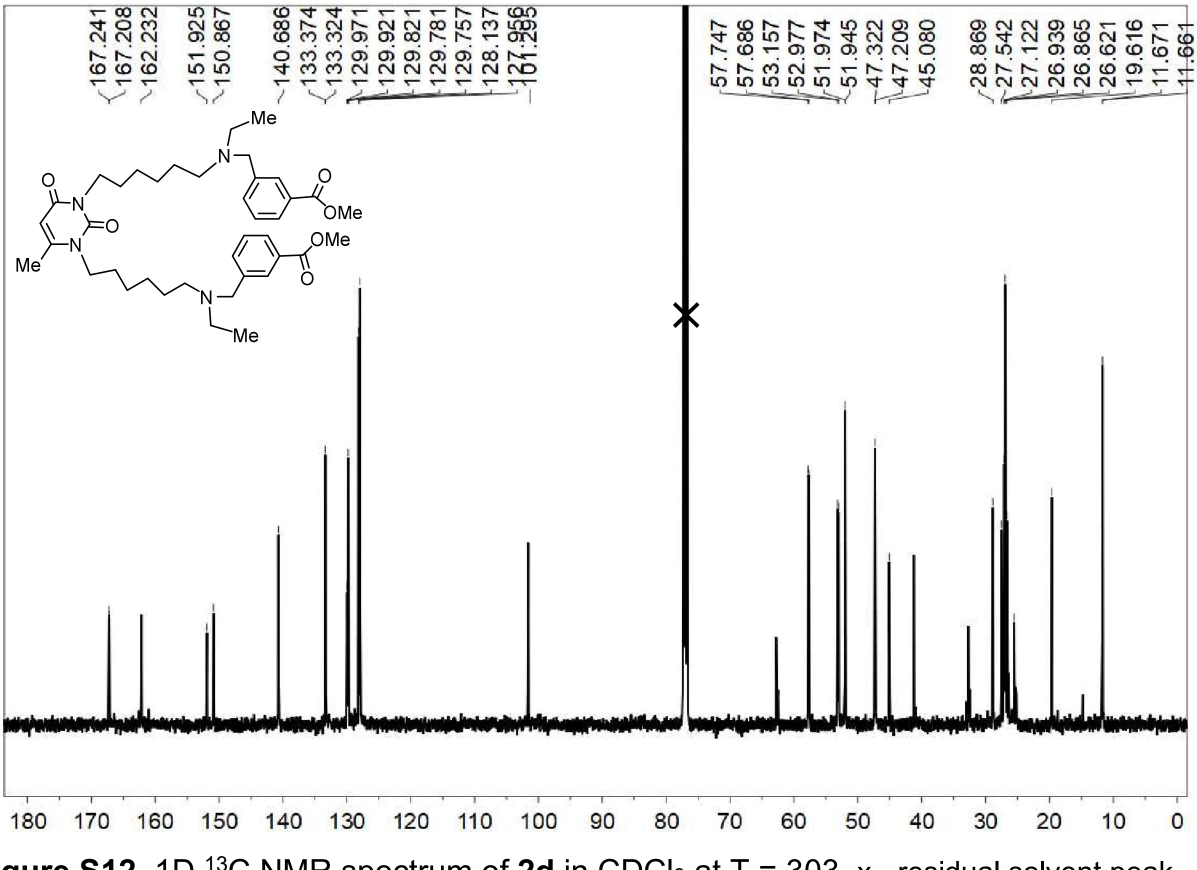

Figure S12. 1D ^{13}C NMR spectrum of **2d** in $CDCl_3$ at T = 303. x - residual solvent peak.

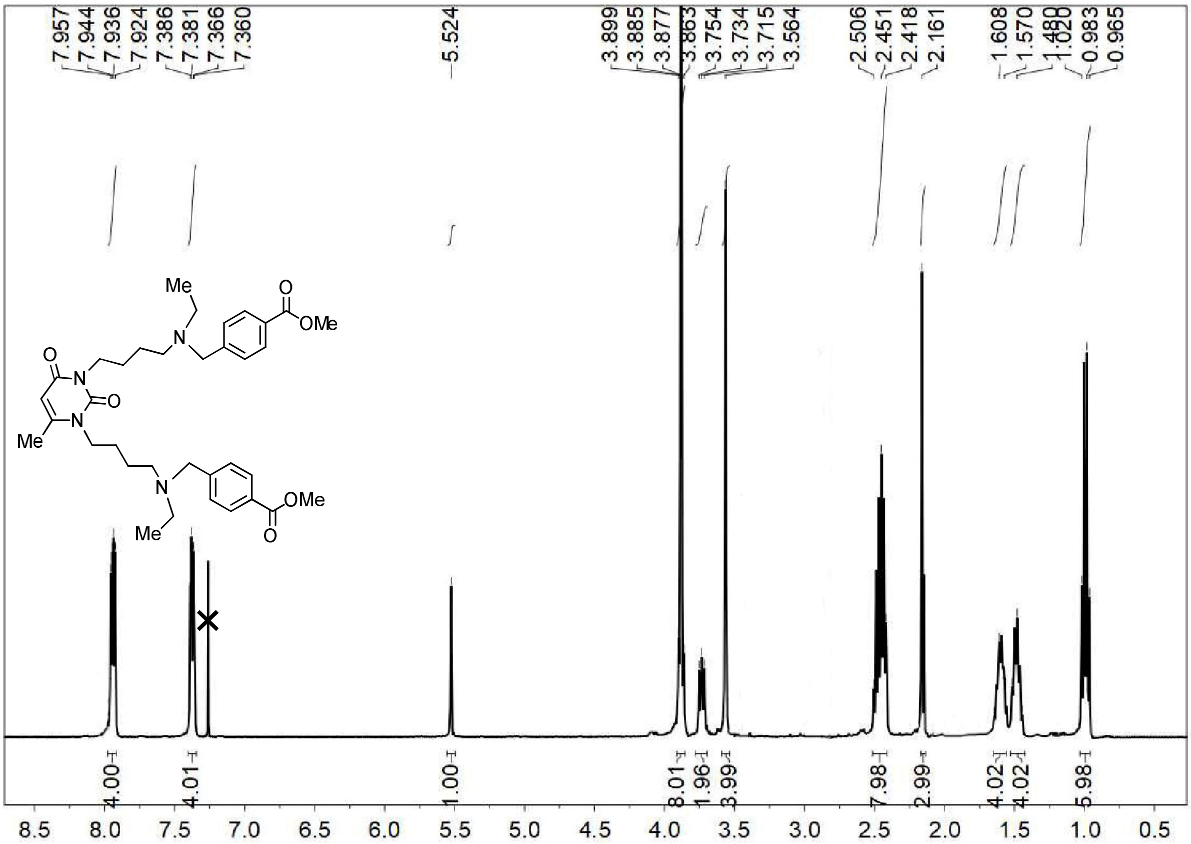

Figure S13. 1D ^{1}H NMR spectrum of **3a** in $CDCl_3$ (400 MHz) at T = 303 K. x - residual solvent peak.

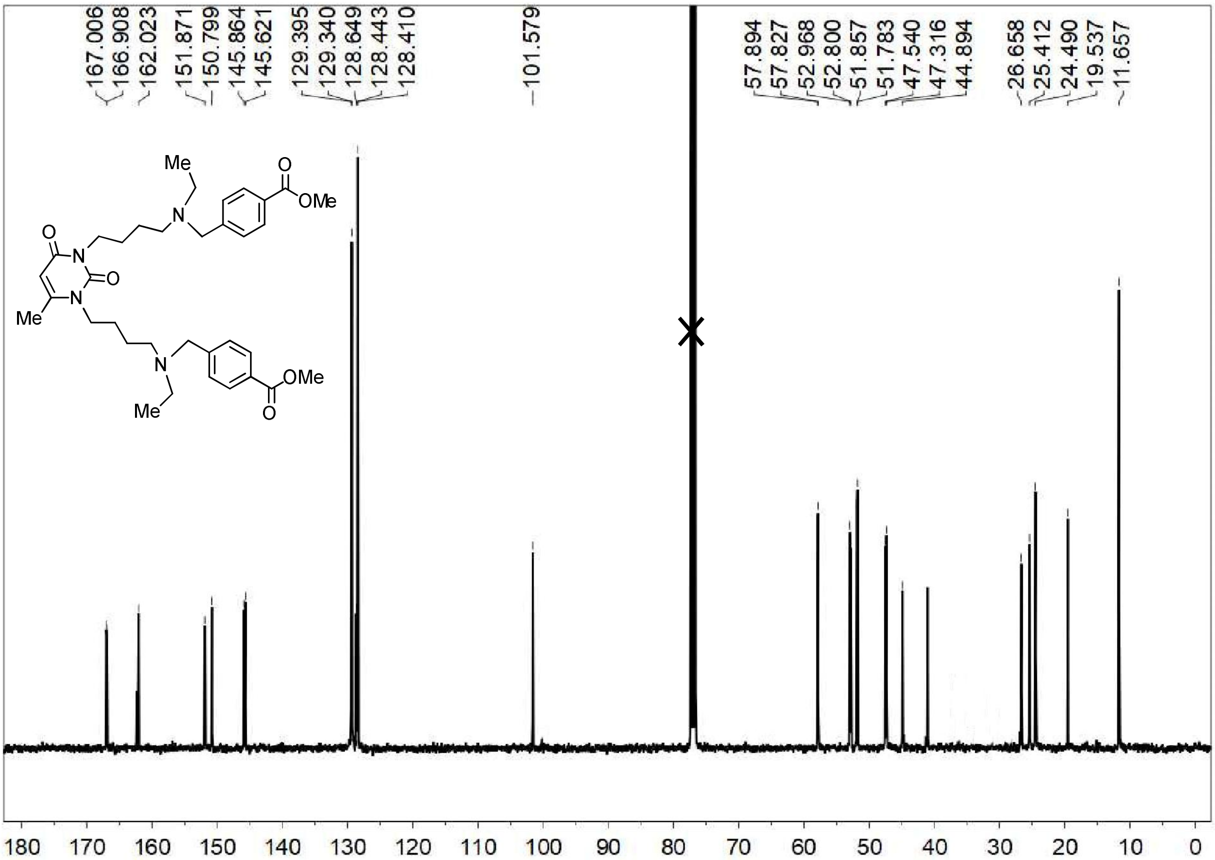

Figure S14. 1D ^{13}C NMR spectrum of **3a** in $CDCl_3$ at T = 303. x - residual solvent peak.

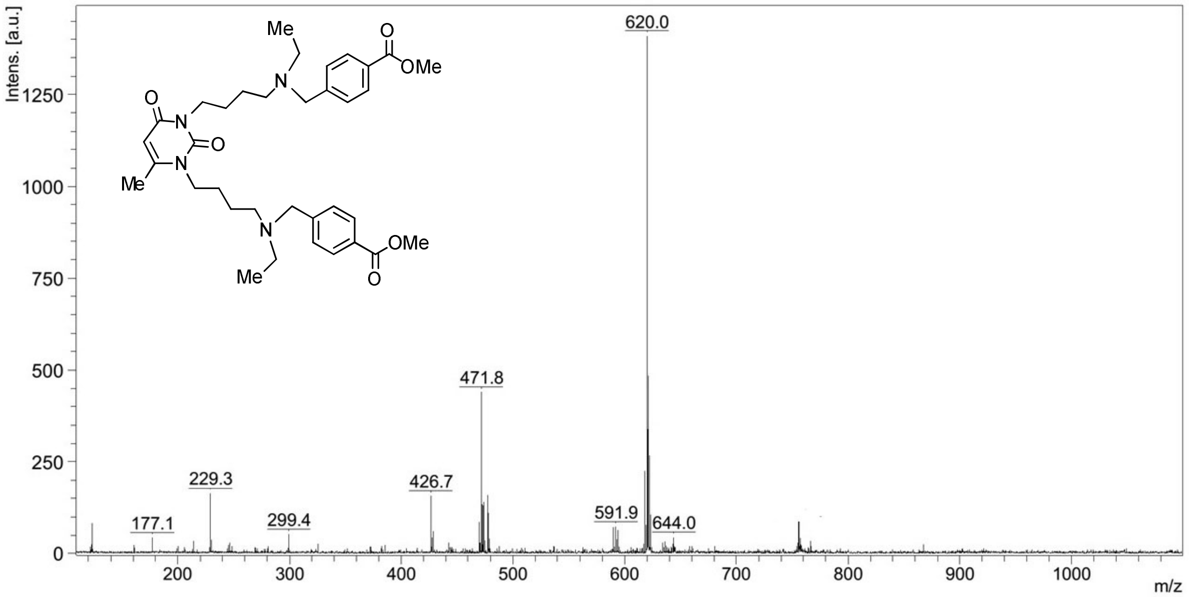

Figure S15. MALDI-TOF mass spectrum of **3a**.

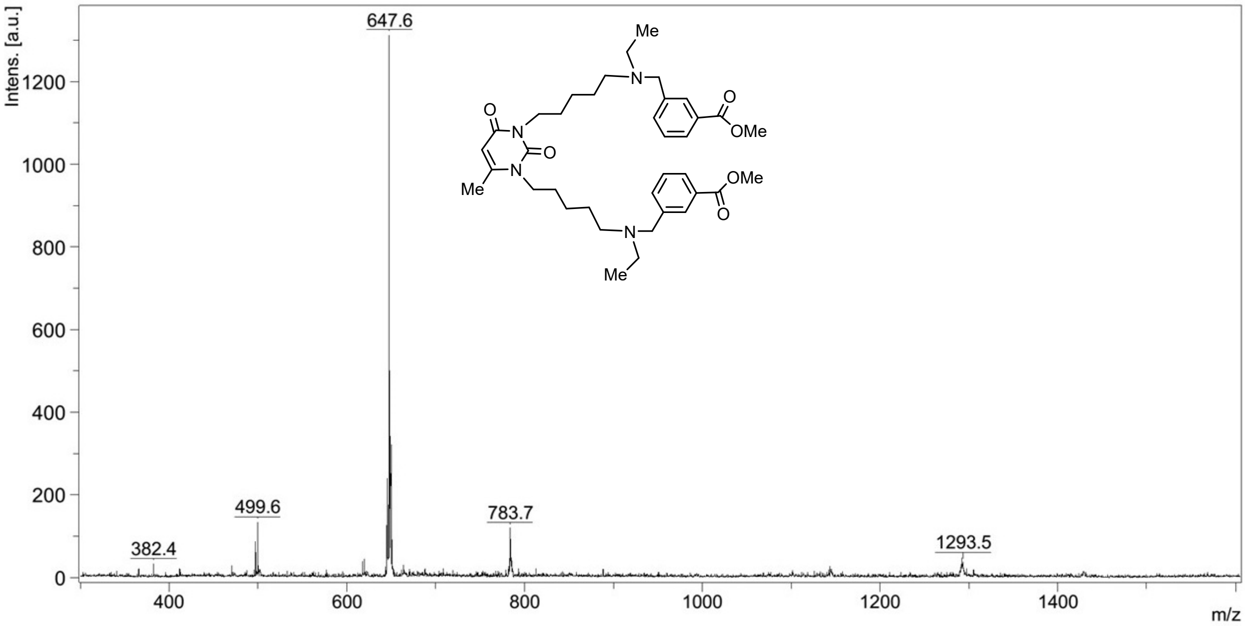

Figure S16. MALDI-TOF mass spectrum of **3b**.

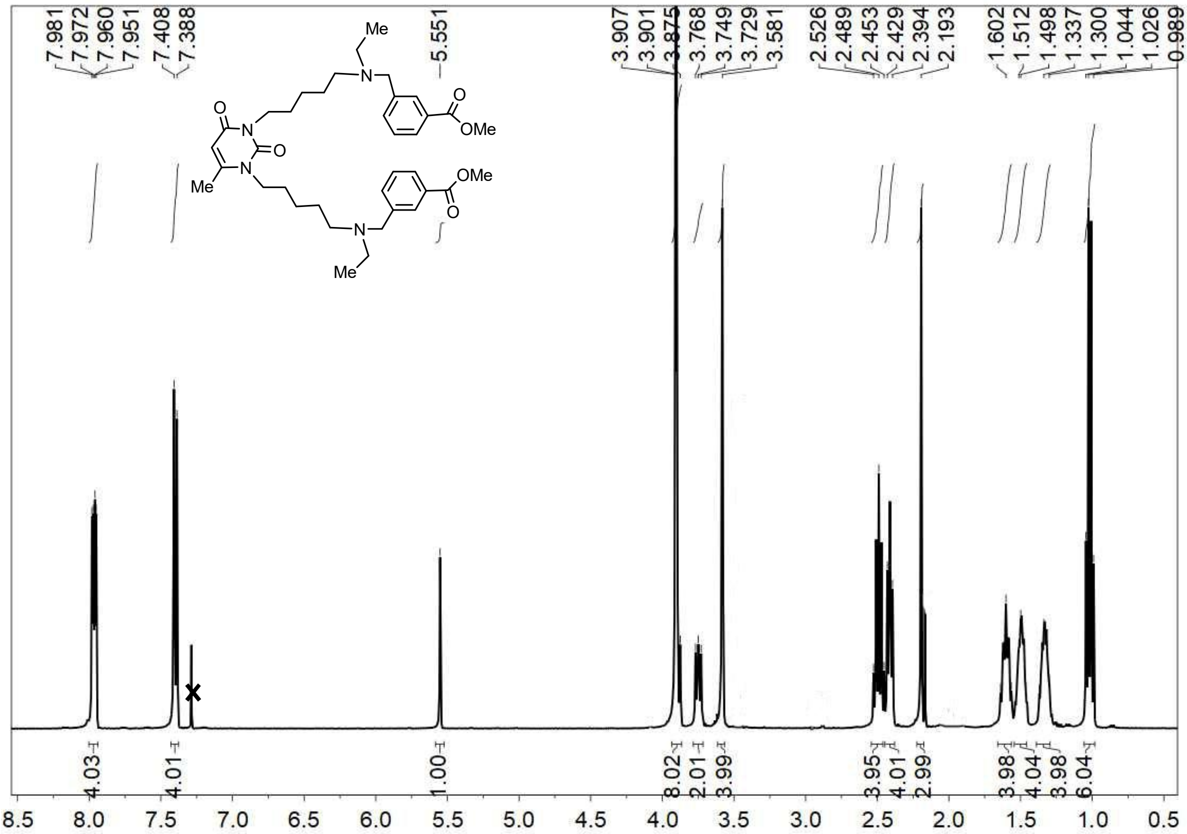

Figure S17. 1D ^{1}H NMR spectrum of **3b** in $CDCl_3$ (400 MHz) at T = 303 K. x - residual solvent peak.

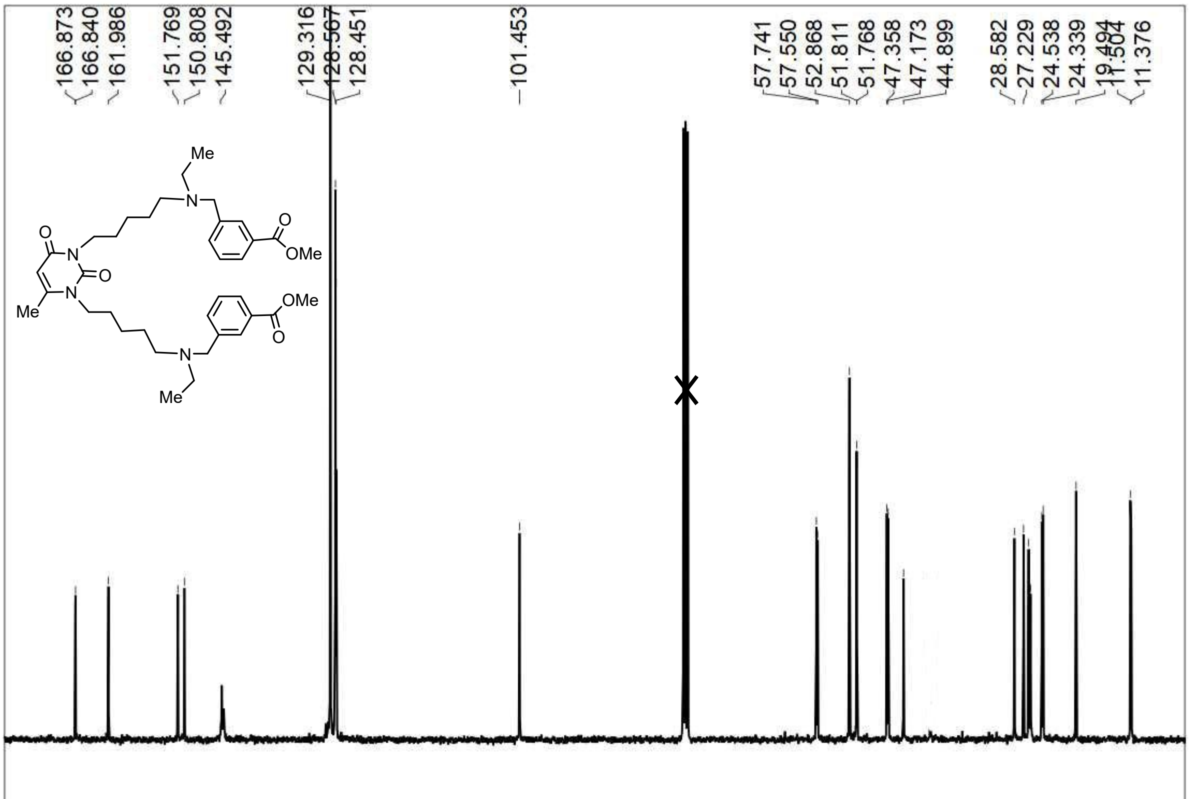

Figure S18. 1D ^{13}C NMR spectrum of **3b** in $CDCl_3$ at T = 303. x - residual solvent peak.

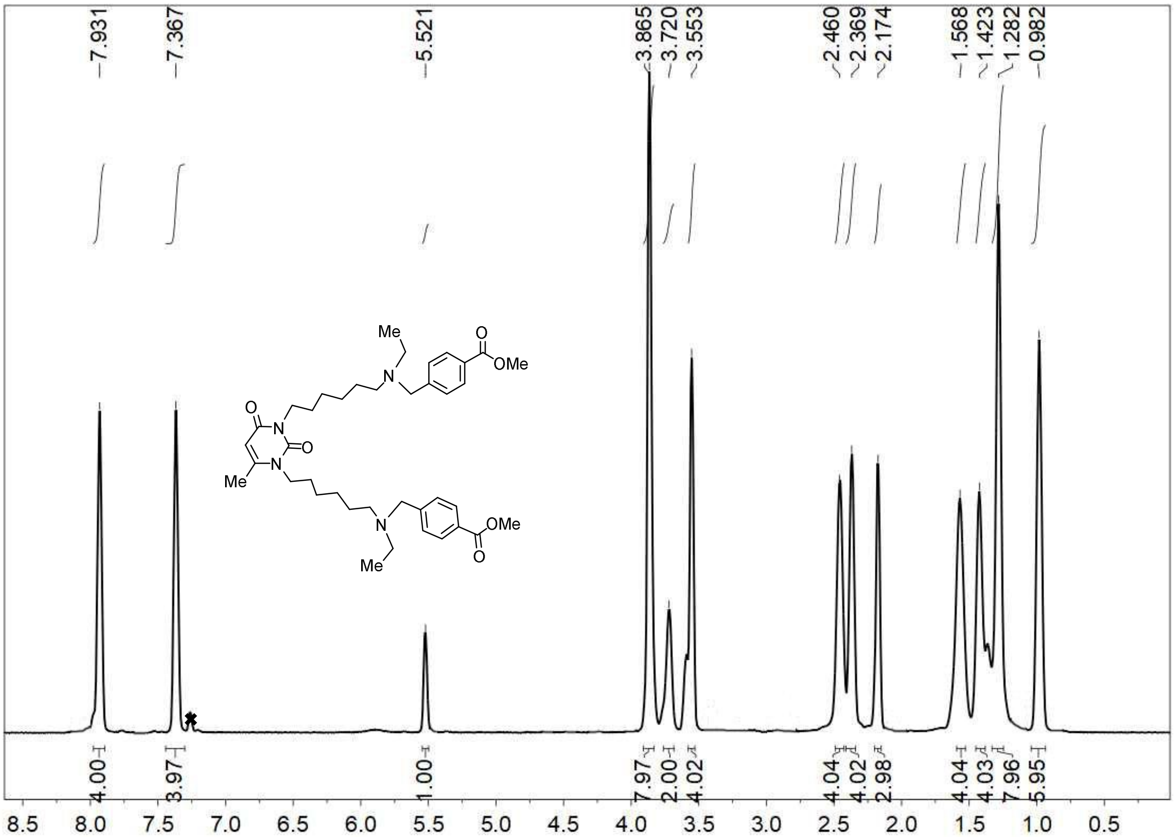

Figure S19. 1D ^{1}H NMR spectrum of **3c** in $CDCl_3$ (400 MHz) at T = 303 K. x - residual solvent peak.

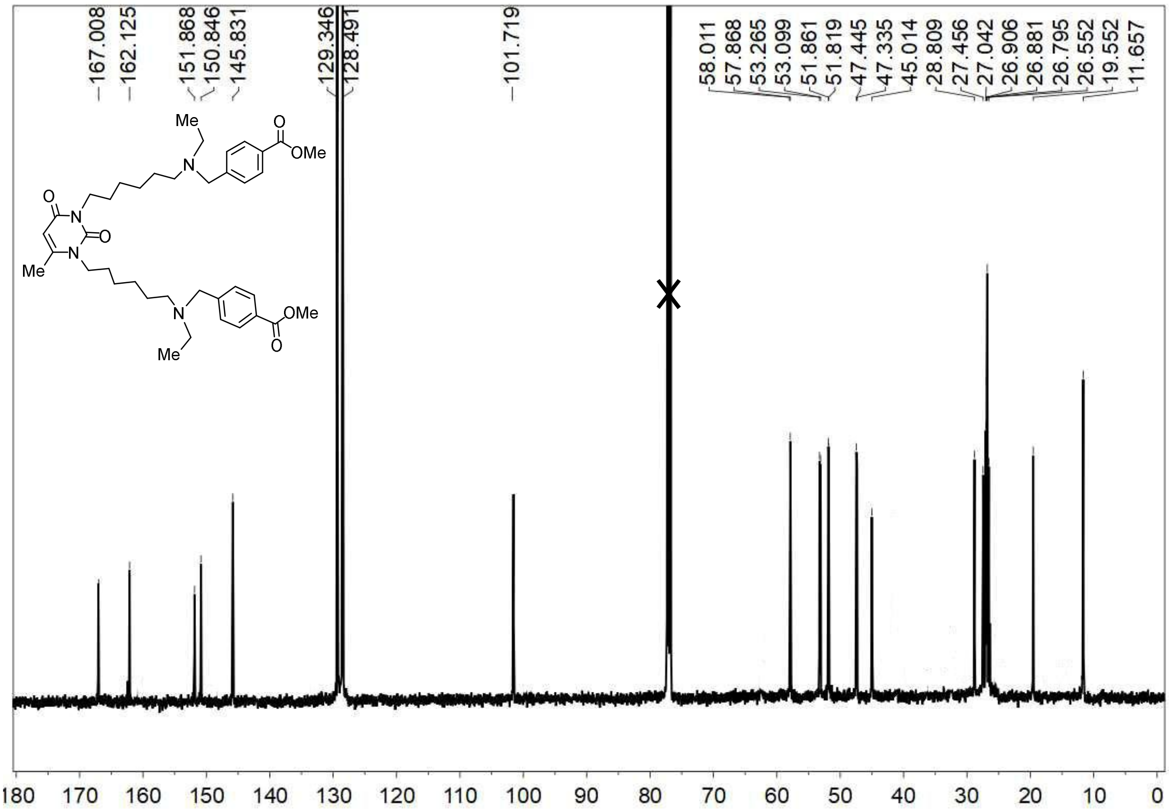

Figure S20. 1D ^{13}C NMR spectrum of **3c** in $CDCl_3$ at T = 303. x - residual solvent peak.

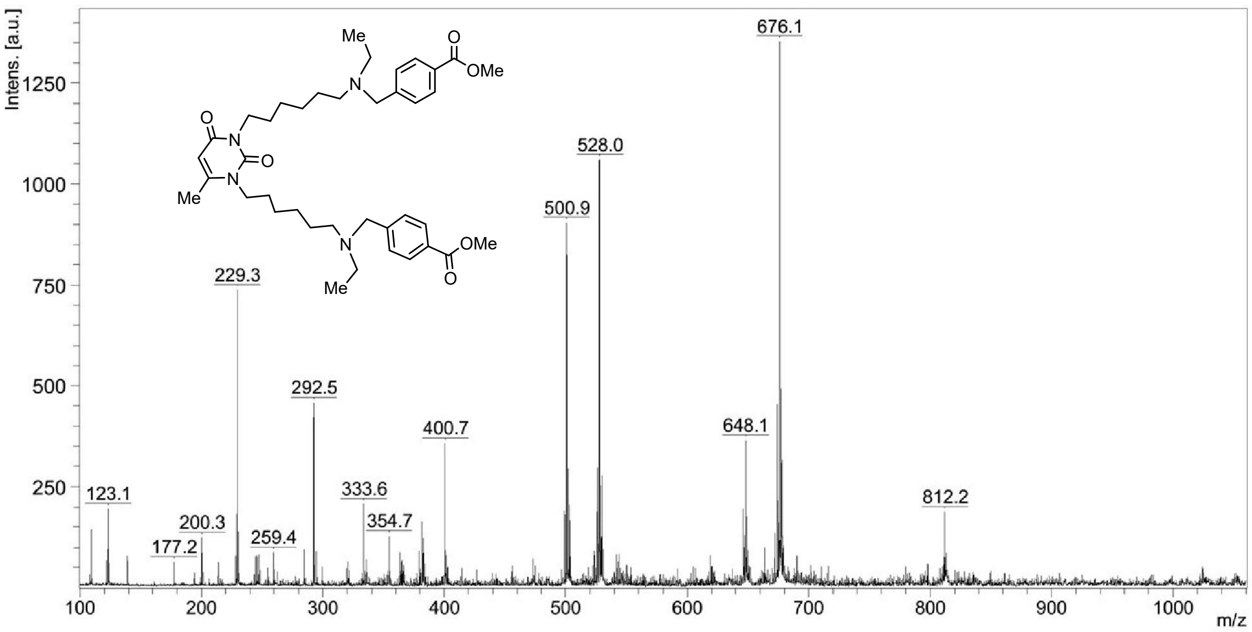

Figure S21. MALDI-TOF mass spectrum of **3c**.

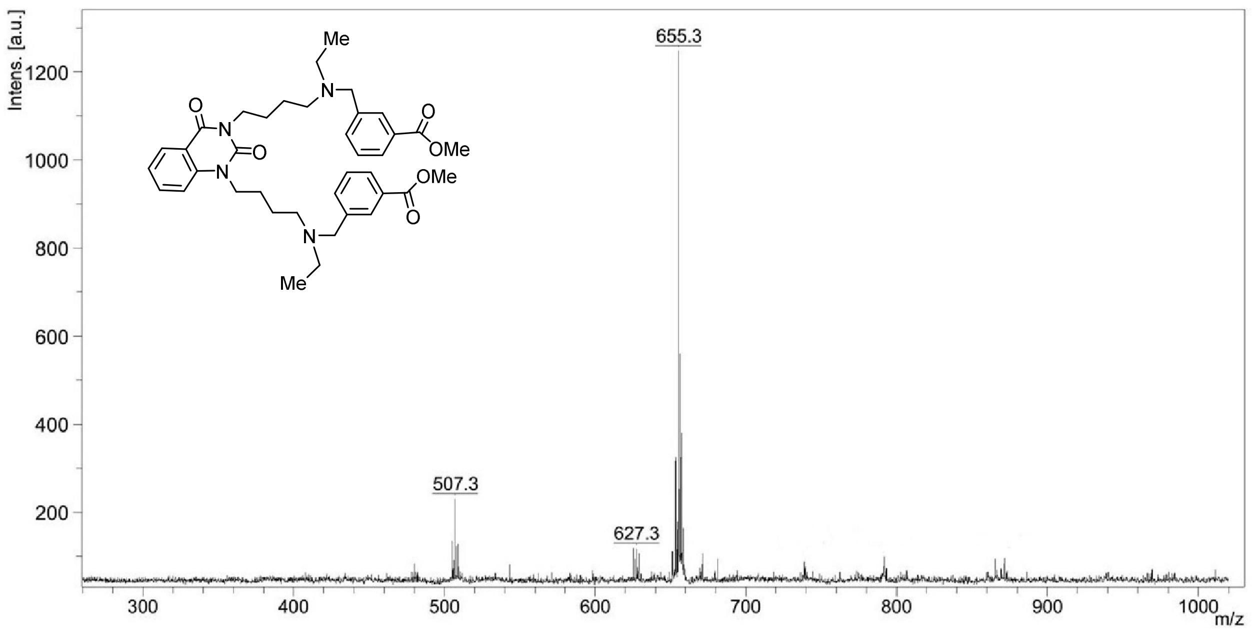

Figure S22. MALDI-TOF mass spectrum of **4a**.

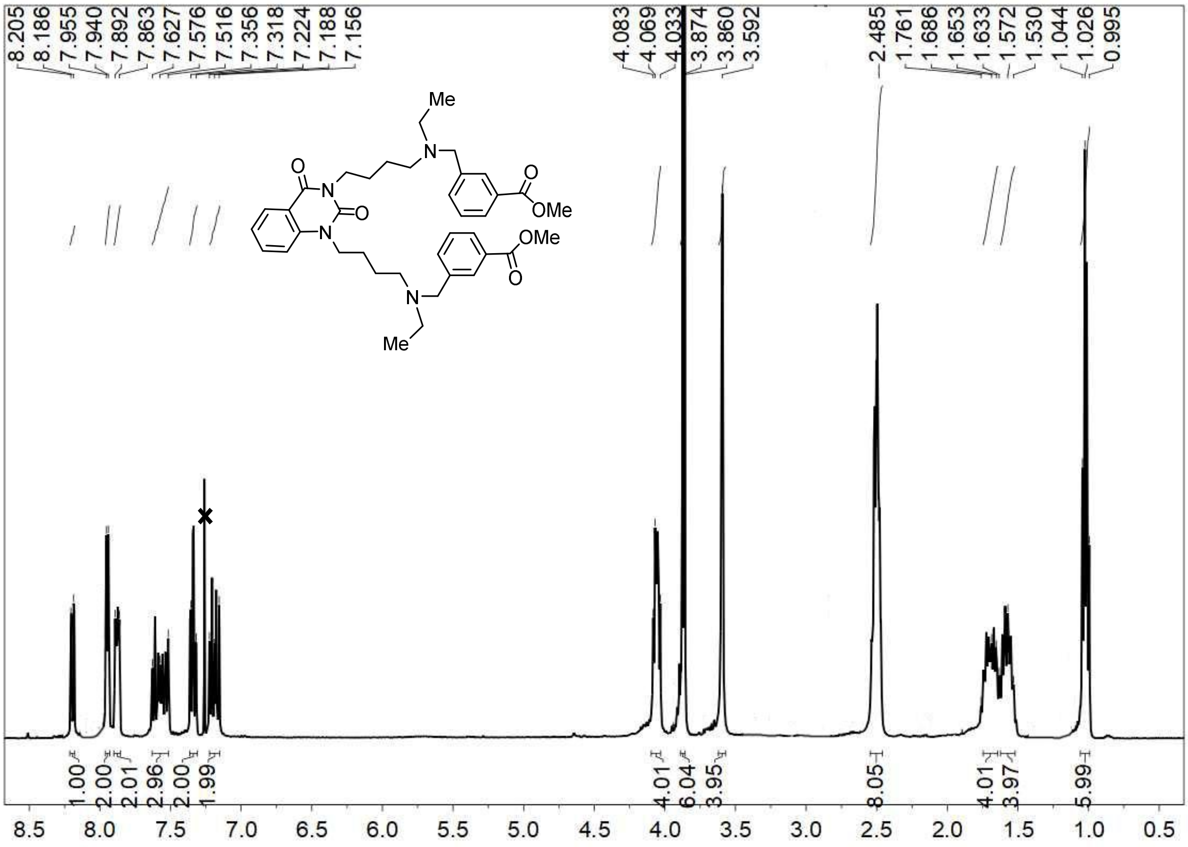

Figure S23. 1D ^{13}C NMR spectrum of **4a** in $CDCl_3$ at T = 303. x - residual solvent peak.

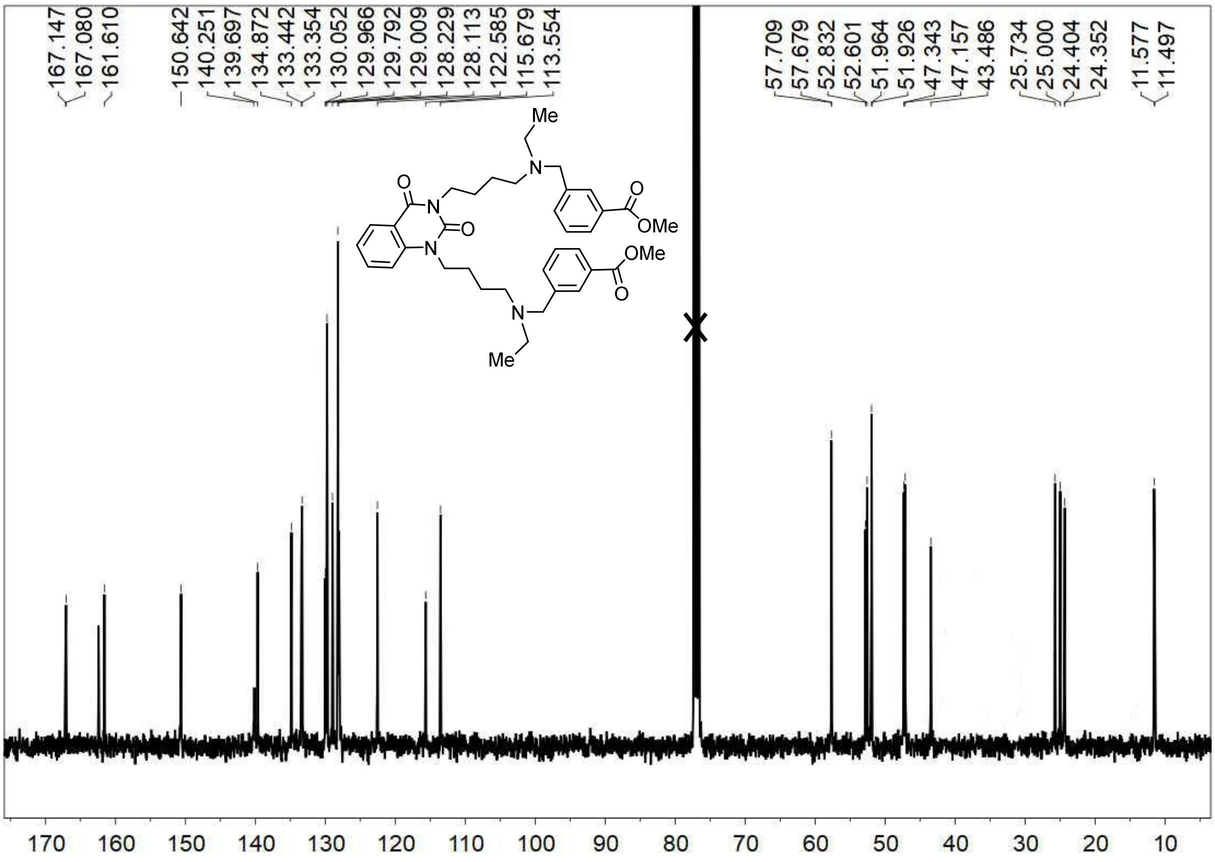

Figure S24. 1D ^{13}C NMR spectrum of **4a** in $CDCl_3$ at T = 303. x - residual solvent peak.

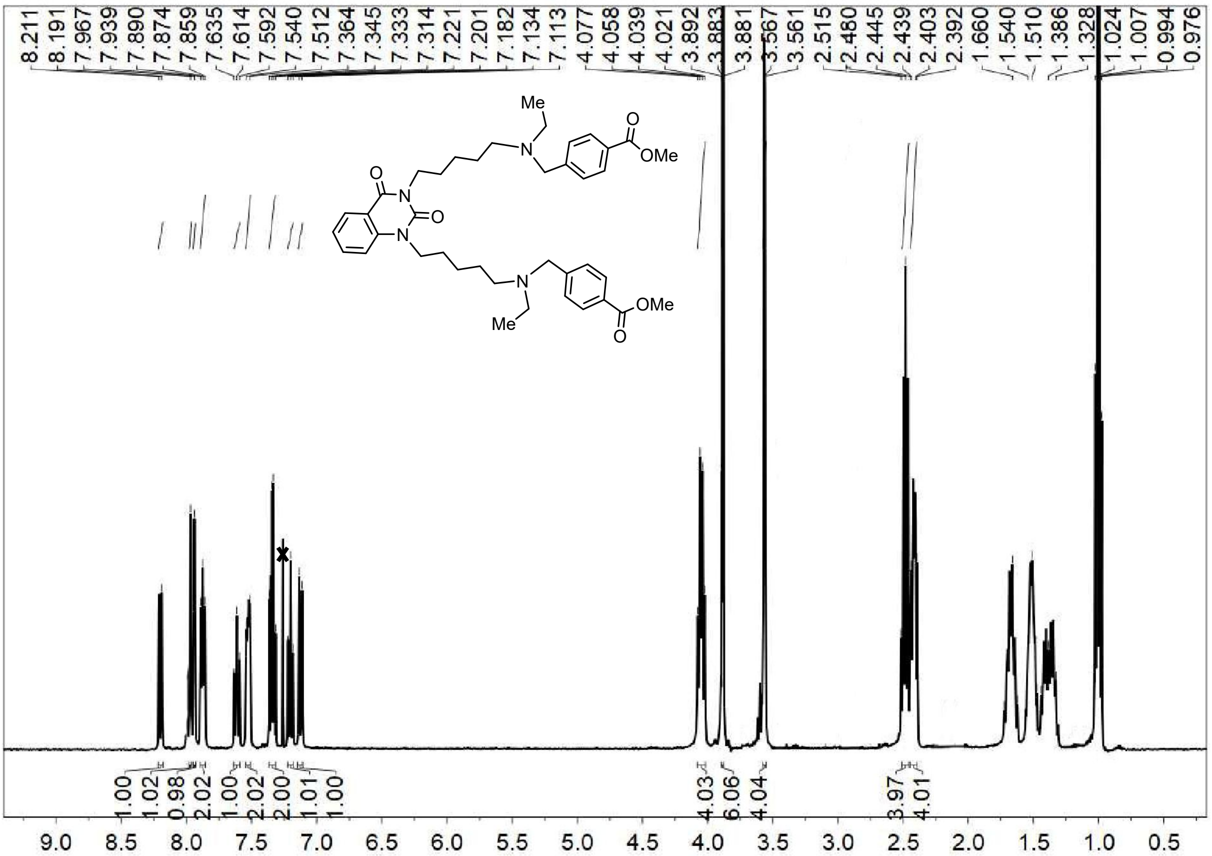

Figure S25. 1D ^{1}H NMR spectrum of **4b** in $CDCl_3$ (400 MHz) at T = 303 K. x - residual solvent peak.

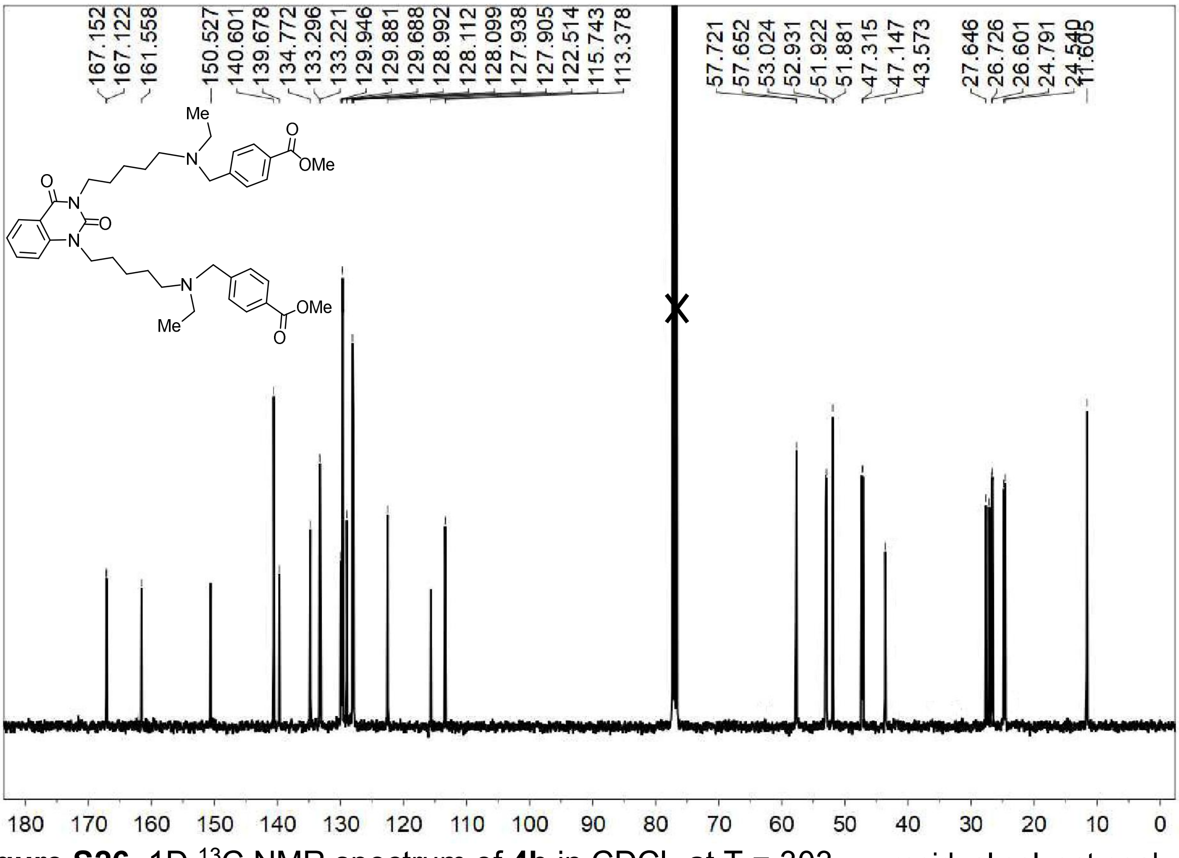

Figure S26. 1D ^{13}C NMR spectrum of **4b** in $CDCl_3$ at T = 303. x - residual solvent peak.

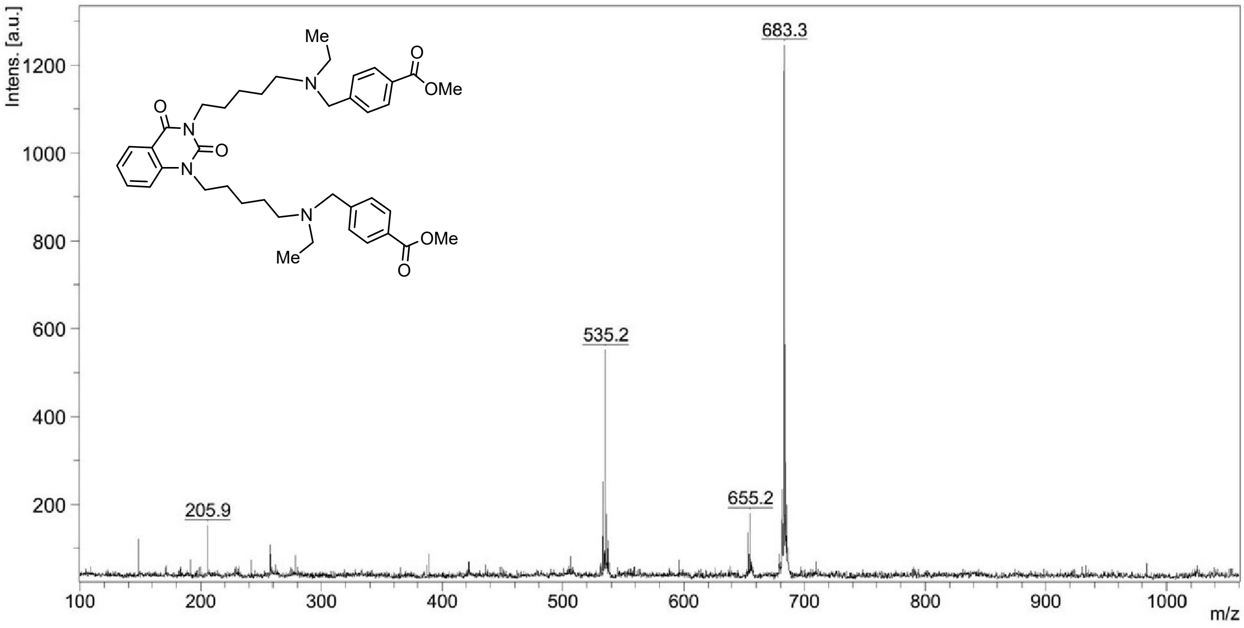

Figure S27. MALDI-TOF mass spectrum of **4b**.

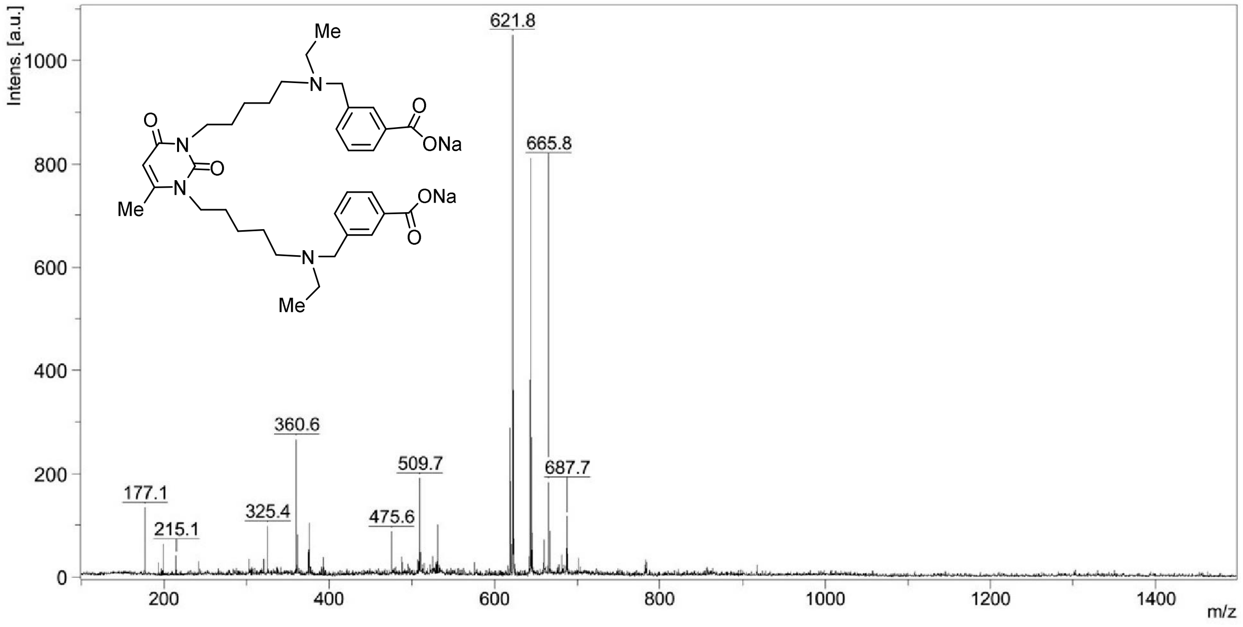

Figure S28. MALDI-TOF mass spectrum of **2e**.

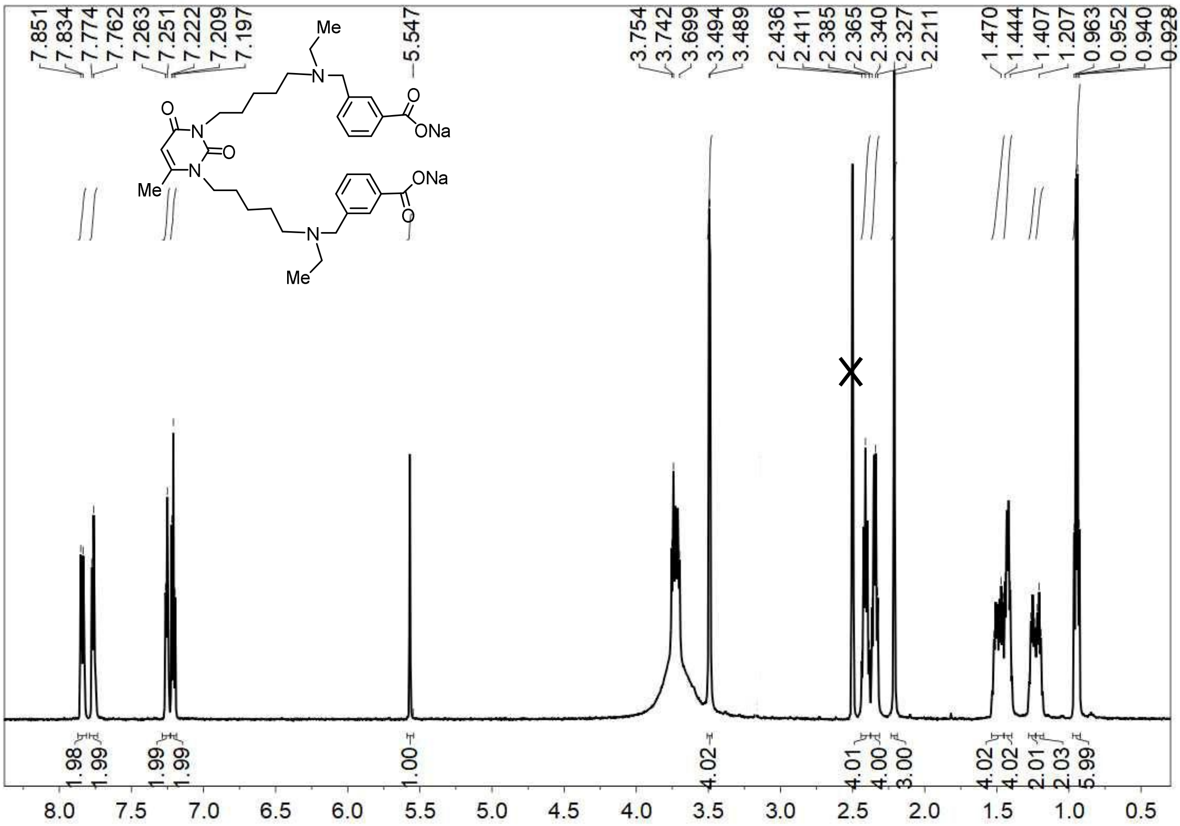

Figure S29. 1D ^{1}H NMR spectrum of **2e** in DMSO-d_6 (600 MHz) at T = 303 K. x - residual solvent peak.

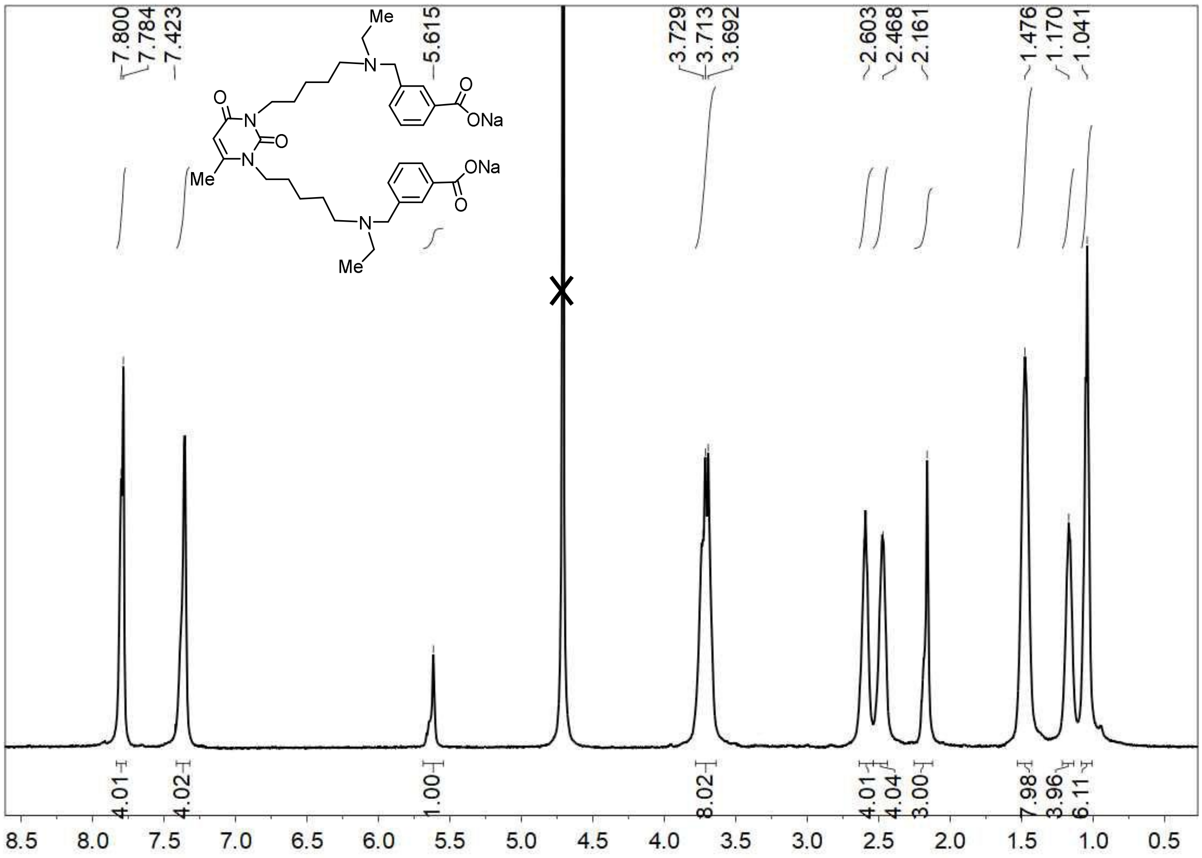

Figure S30. 1D ^{1}H NMR spectrum of **2e** in D_2O (600 MHz) at T = 303 K. x - residual solvent peak.

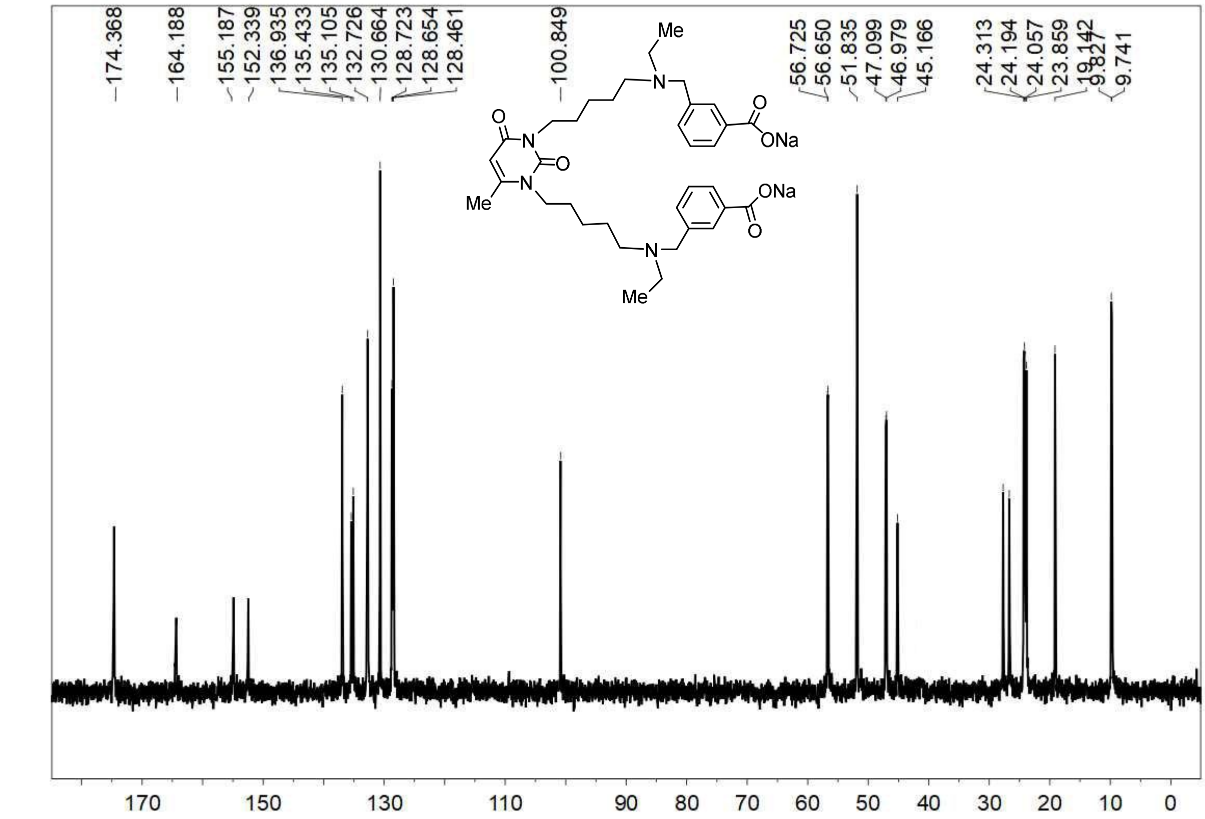

Figure S31. 1D ^{13}C NMR spectrum of **2e** in D_2O (150 MHz) at T = 303 K.

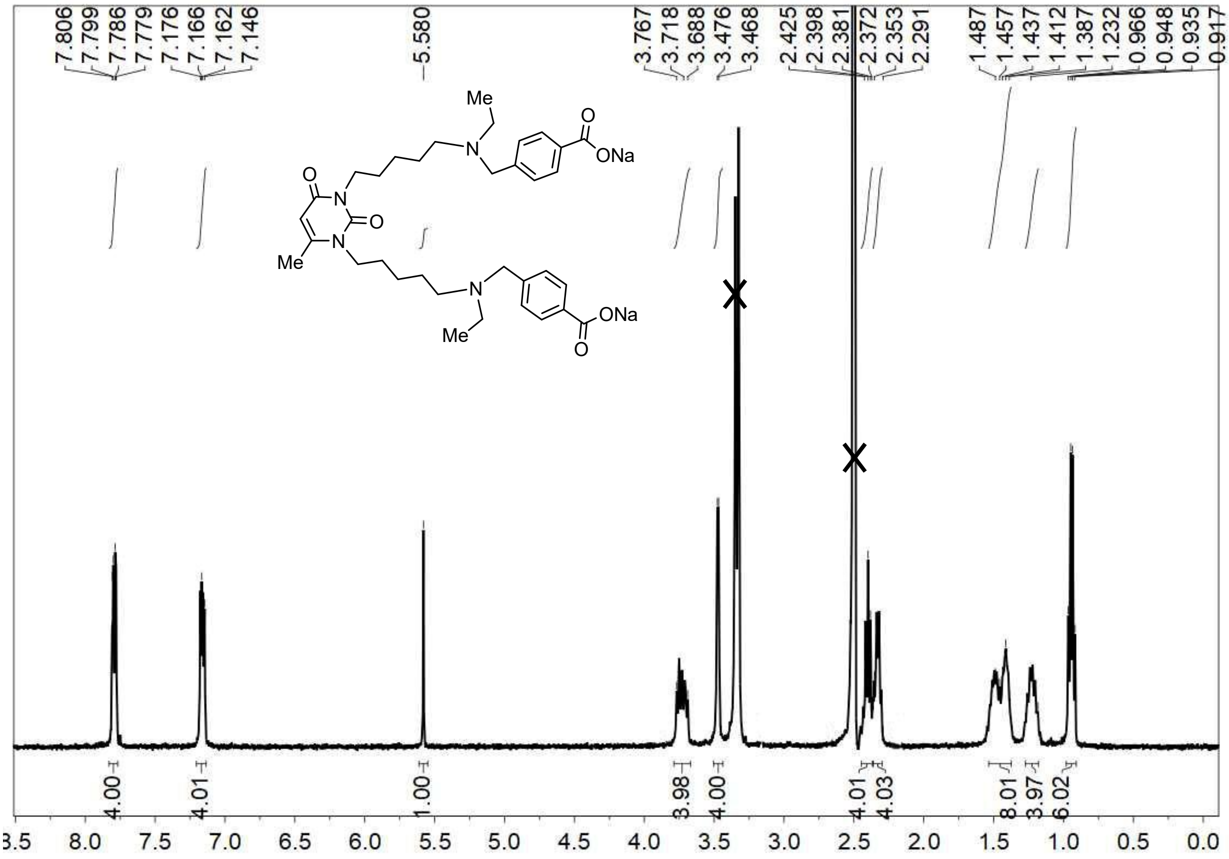

Figure S32. 1D ^{1}H NMR spectrum of **3d** in DMSO-d_6 (600 MHz) at T = 303 K. x - residual solvent peak.

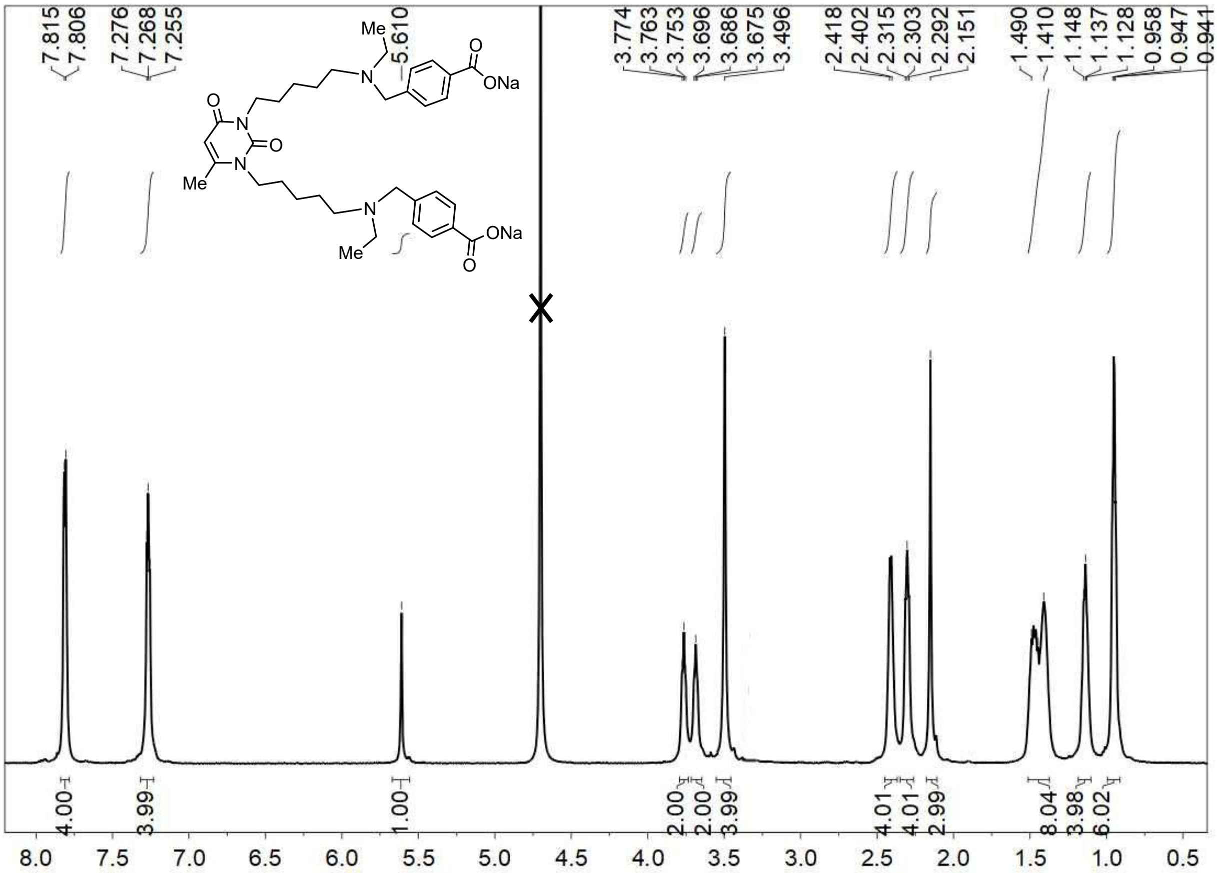

Figure S33. 1D ^{1}H NMR spectrum of **3d** in D_2O (600 MHz) at T = 303 K. x - residual solvent peak.

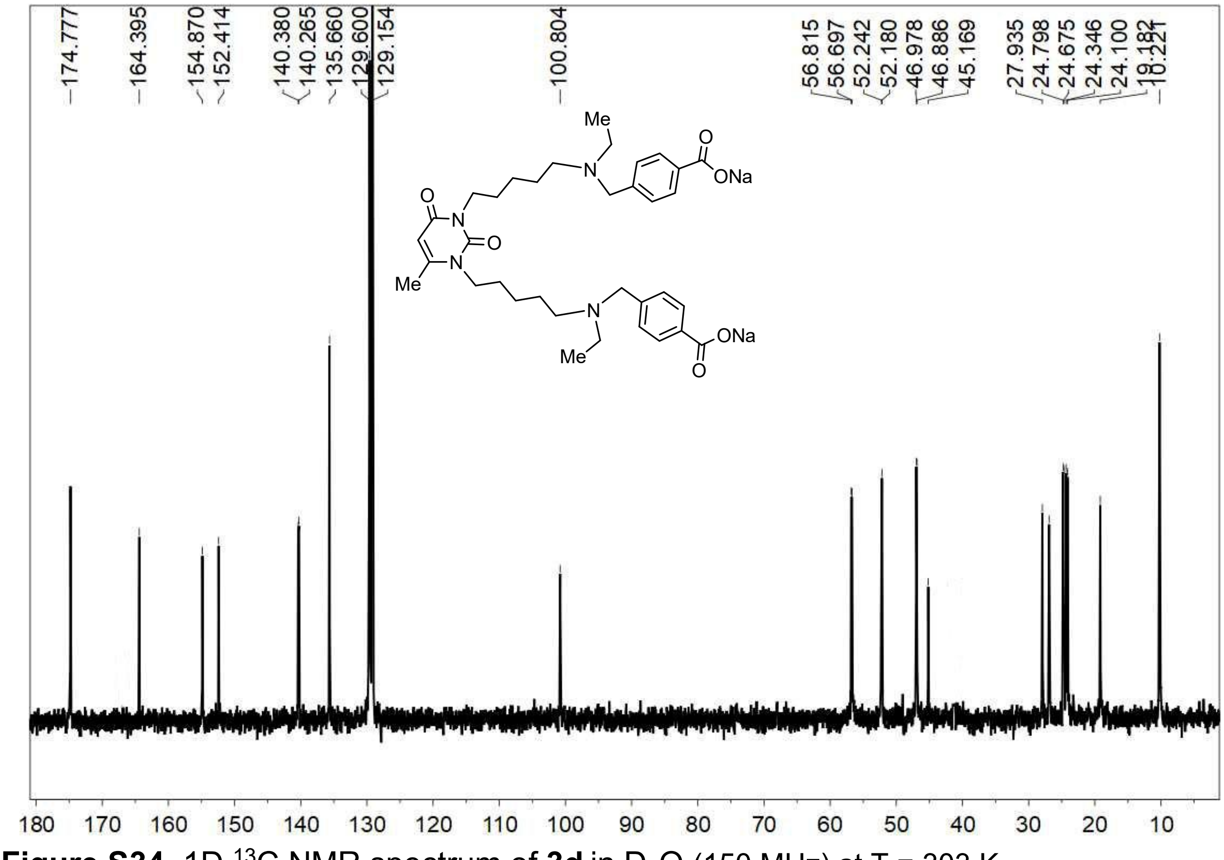

Figure S34. 1D ^{13}C NMR spectrum of **3d** in D_2O (150 MHz) at T = 303 K.

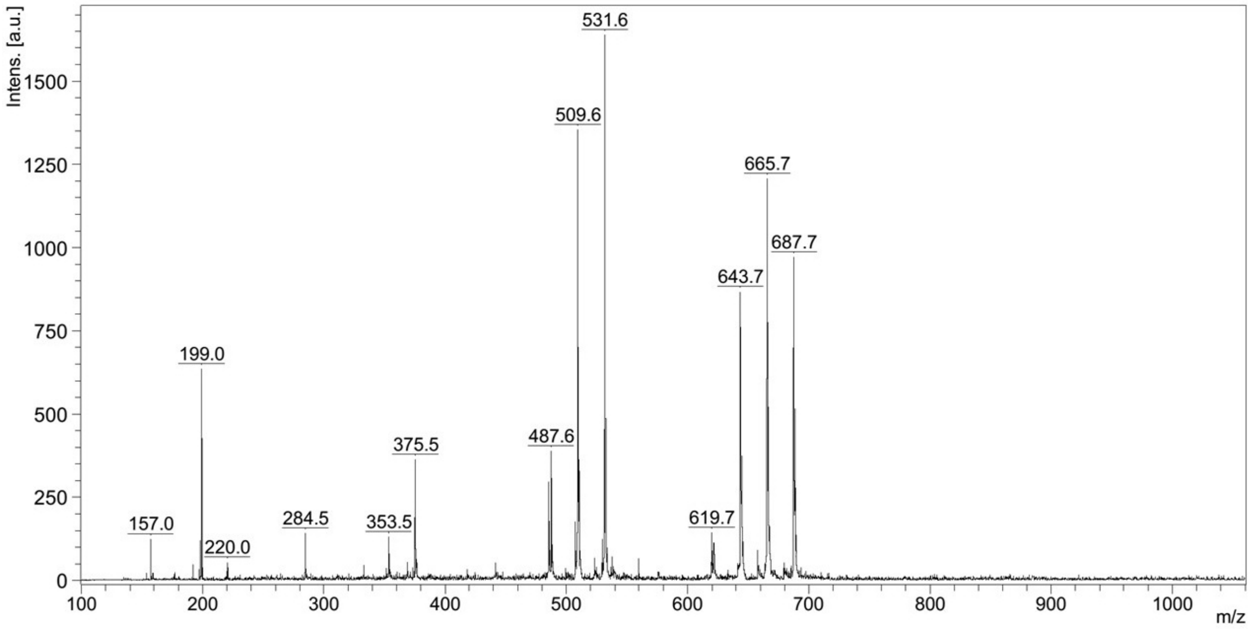

Figure S35. MALDI-TOF mass spectrum of **3d**.

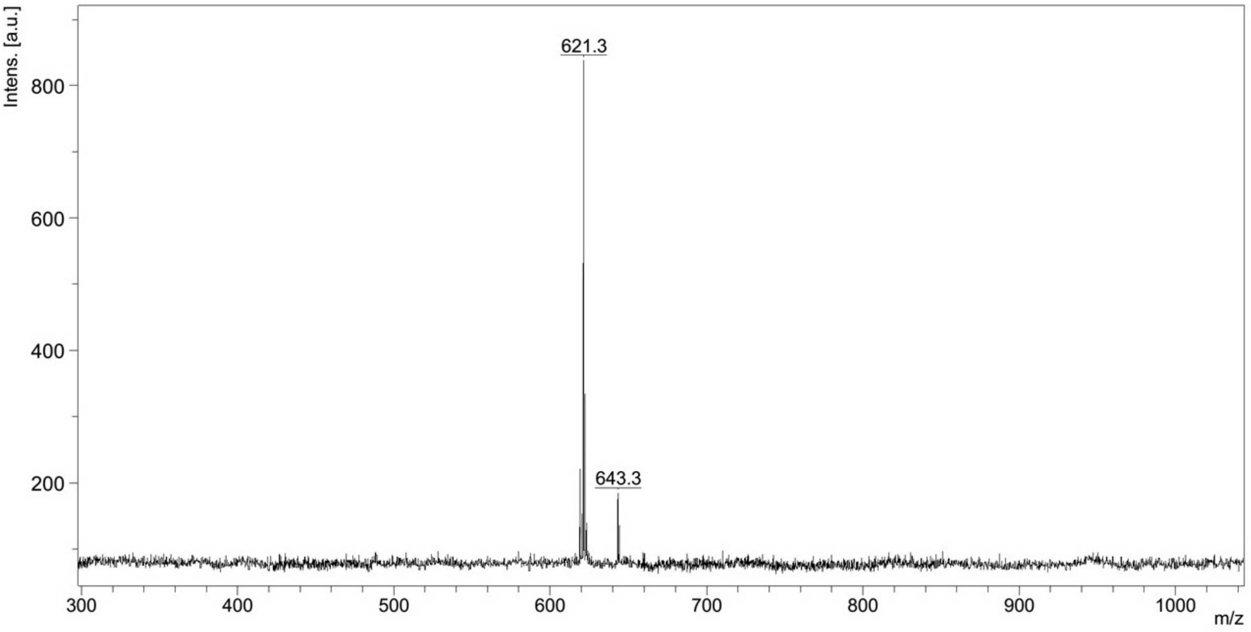

Figure S36. MALDI-TOF mass spectrum of **2f**.

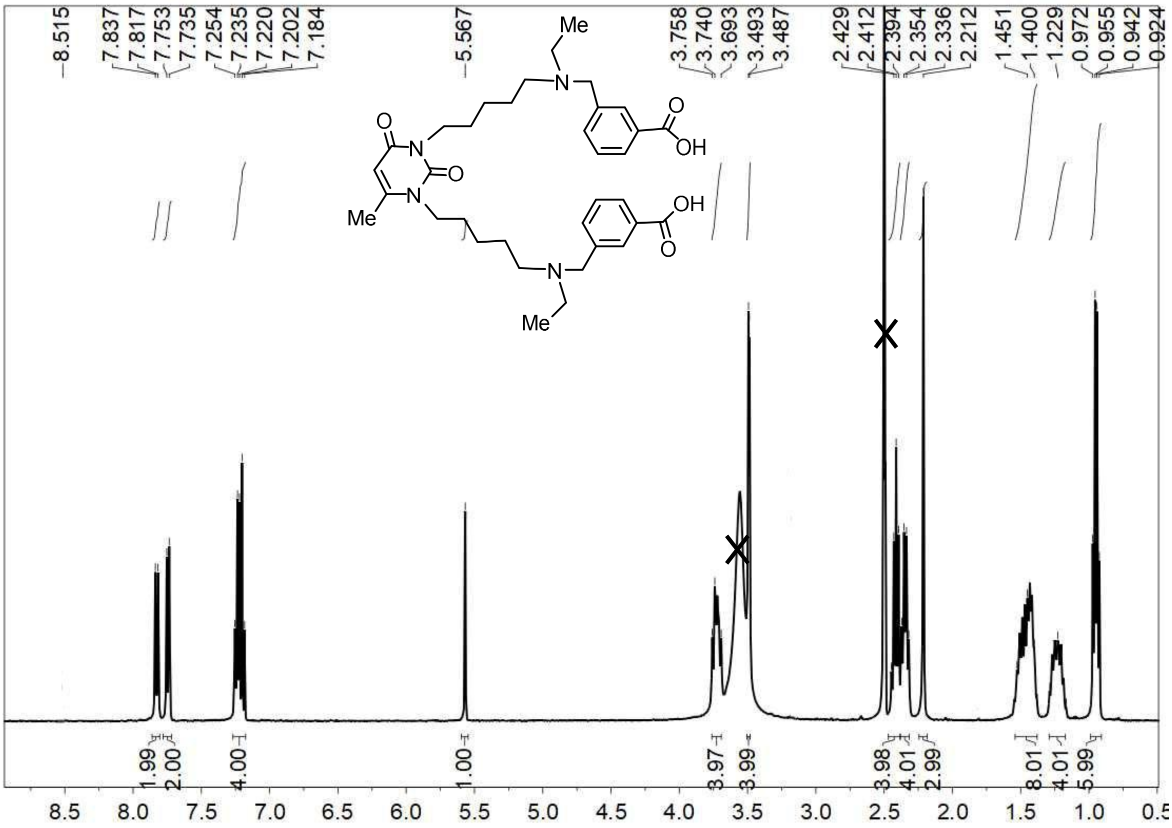

Figure S37. 1D ^{1}H NMR spectrum of **2f** in DMSO-d_6 (400 MHz) at T = 303 K. x - residual solvent peak.

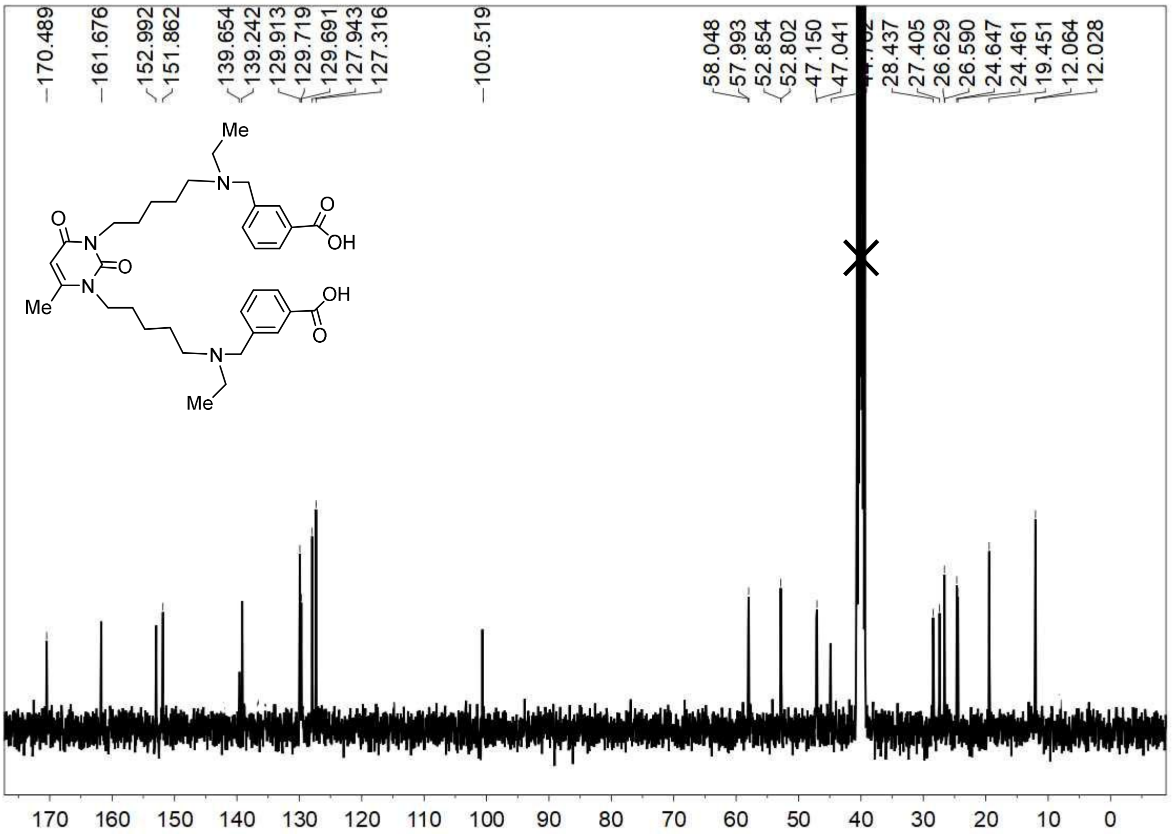

Figure S38. 1D ^{13}C NMR spectrum of **2f** in DMSO-d_6 (100 MHz) at T = 303 K.

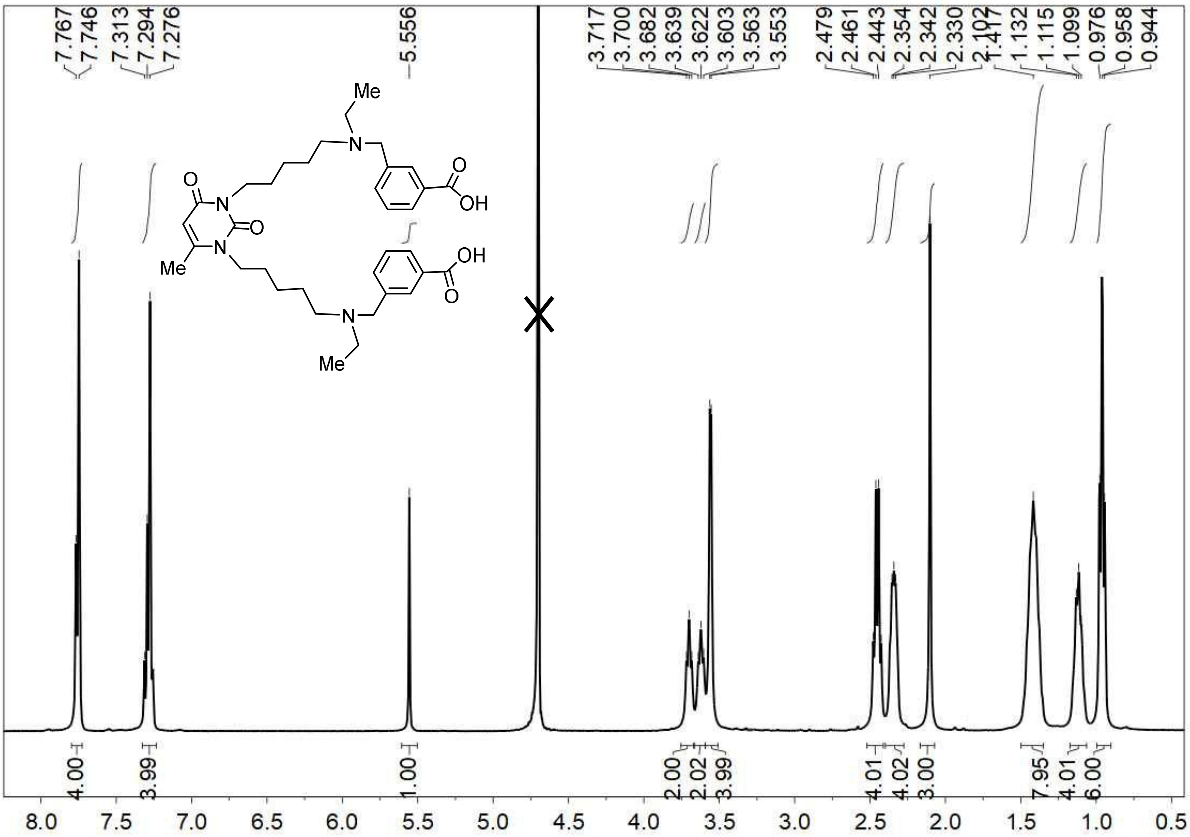

Figure S39. 1D ^{1}H NMR spectrum of **2f** in D_2O (600 MHz) at T = 303 K. x - residual solvent peak.

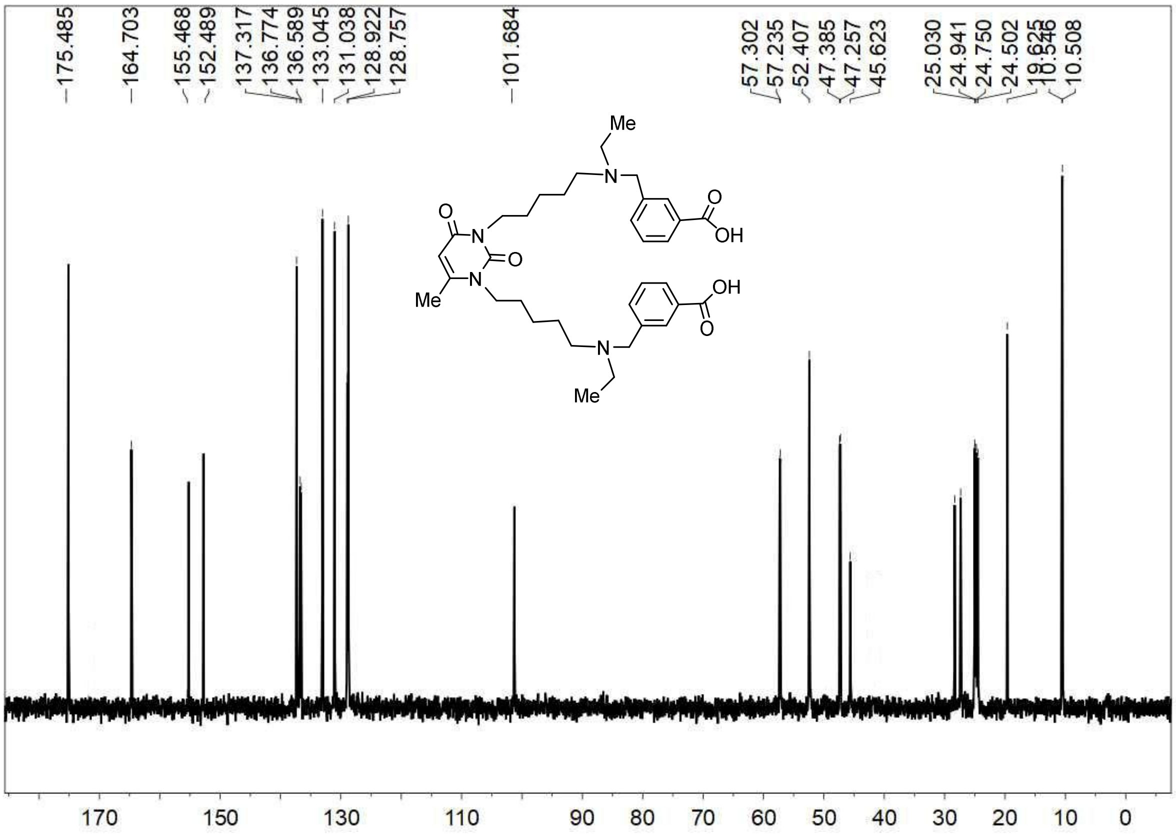

Figure S40. 1D ^{13}C NMR spectrum of **2f** in D_2O (150 MHz) at T = 303 K.

NMR study of the stability of compound 2c

The compound **2c** was dissolved in ethanol and then in 2 ml of 0.1M sodium phosphate buffer (pH=8.0) at the final concentration of 0.1 mM. Then 2.0 units of hAChE was added. The final concentration of ethanol in the buffer was 0.1% vol. The compound **2c** was incubated with hAChE overnight at 25°C. The same volume of buffer containing compound **2c** at the concentration of 0.1 mM but without AChE was used as a control. After the end of incubation, the buffer was filtered out of AChE using a centricon-30 ultrafiltration micro-concentrator from Amicon (Millipore Corporation, Billerica, MA, USA). Samples of the phosphate buffer were evaporated in vacuum, 1 ml of deuterated chloroform was added to the residue, the mixture was filtered and transferred to a 5 mm ampoule to register NMR spectra.

A marker of the presence of an ester, in particular a methoxycarbonyl group is a signal of a methyl radical of the methoxycarbonyl fragment located in the region of 3.90 ppm as a singlet. In the case of complete hydrolysis of the ester group to a carboxylic one, this singlet will be absent in the spectrum of the hydrolysis product, as is the case, for example, in the ^{1}H NMR spectra of sodium salt **2e** and acid **2f**. These compounds were obtained as a result of five-hour boiling of ester **2c** in an alkali solution. In the case of partial hydrolysis of ester groups, a set of resonance signals of both the ester, *m*-methoxycarbonylbenzylethylamino fragment and the acid, *m*-carboxylbenzylethylamino fragment, would be observed in the spectrum of hydrolysis products.

Figure S41 shows the spectrum of ester **2c** and its description. Also, this spectrum is shown in the above figure S7.

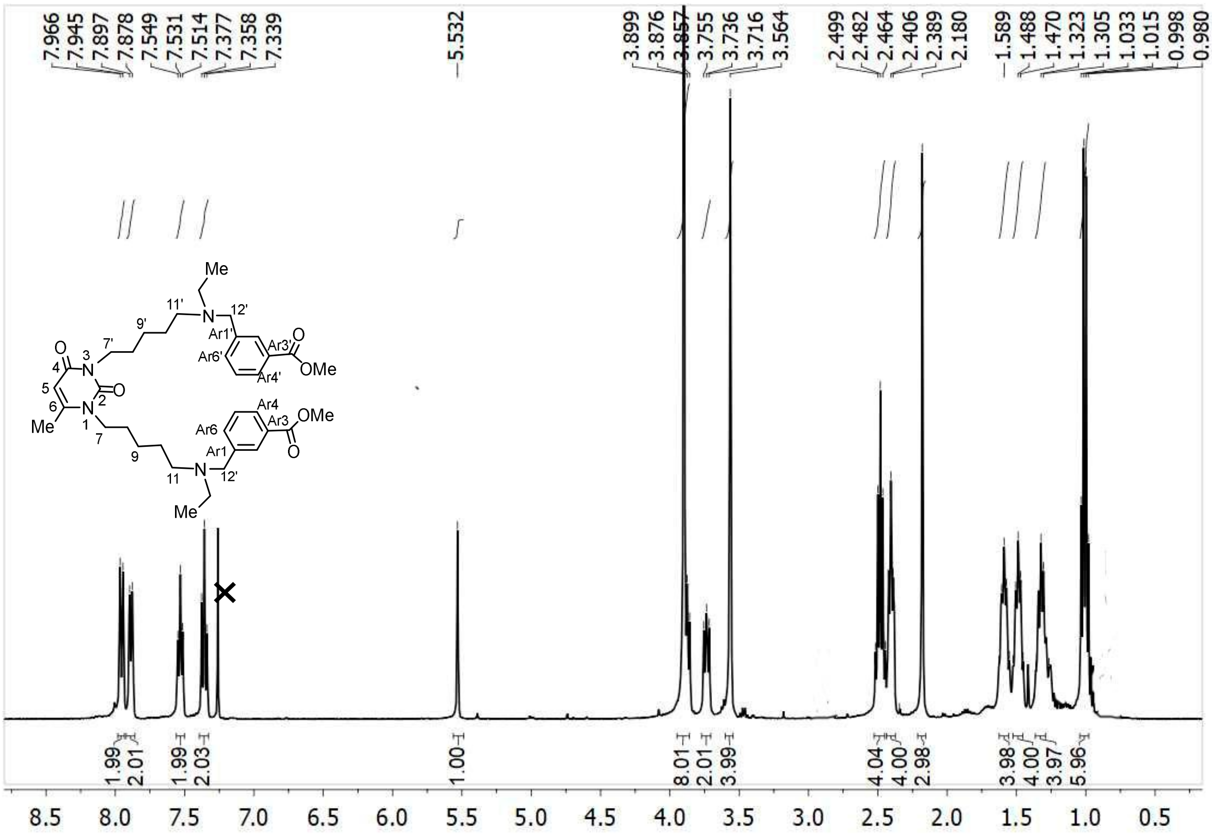

Figure S41. 1D ^{1}H NMR spectrum of **2c** in $CDCl_3$ (600 MHz) at T = 303 K. x - residual solvent peak.

1,3-Bis[5-(m-methoxycarbonylbenzylethylamino)pentyl]-6-methyluracil (***2c***): ^{1}H NMR ($CDCl_3$, 400 MHz) δ 7.97, 7.95 (both s, 1H each, 2H, Ar2H, Ar2'H), 7.90-7.88 (both m, 2H each, 4H, Ar5H, Ar5'H, Ar4H, Ar4'H), 7.38-7.34 (m, 2H, Ar6H, Ar6'H), 5.53 (s, 1H, C5H), 3.90 (br. s, 6H,

2COOC$\underline{H_3}$), 3.88-(3.86 m, 2H, N3C7'H_2), 3.76-3.72 (m, 2H, N1C7H_2), 3.56 (br. s, 4H, C12CH_2, C12'CH_2), 2.52-2.46, 2.45-2.39 (both m, 4H each, 8H, C11CH_2, C11'CH_2, 2NC$\underline{H_2}$$CH_3$), 2.18 (s, 3H, C6$CH_3$), 1.63-1.55, 1.52-1.45, 1.36-1.27 (all m, 4H each, 12H, C8CH_2, C9CH_2, C10CH_2, C8'CH_2, C9'CH_2, C10'CH_2), 1.03-0.98 (m, 6H, 2NCH_2C$\underline{H_3}$).
With the complete hydrolysis of ester **2c**, acid **2f** should be formed. Figure 42 shows ^{1}H NMR spectrum of acid **2f**, the same spectrum is shown above in Figure S22.

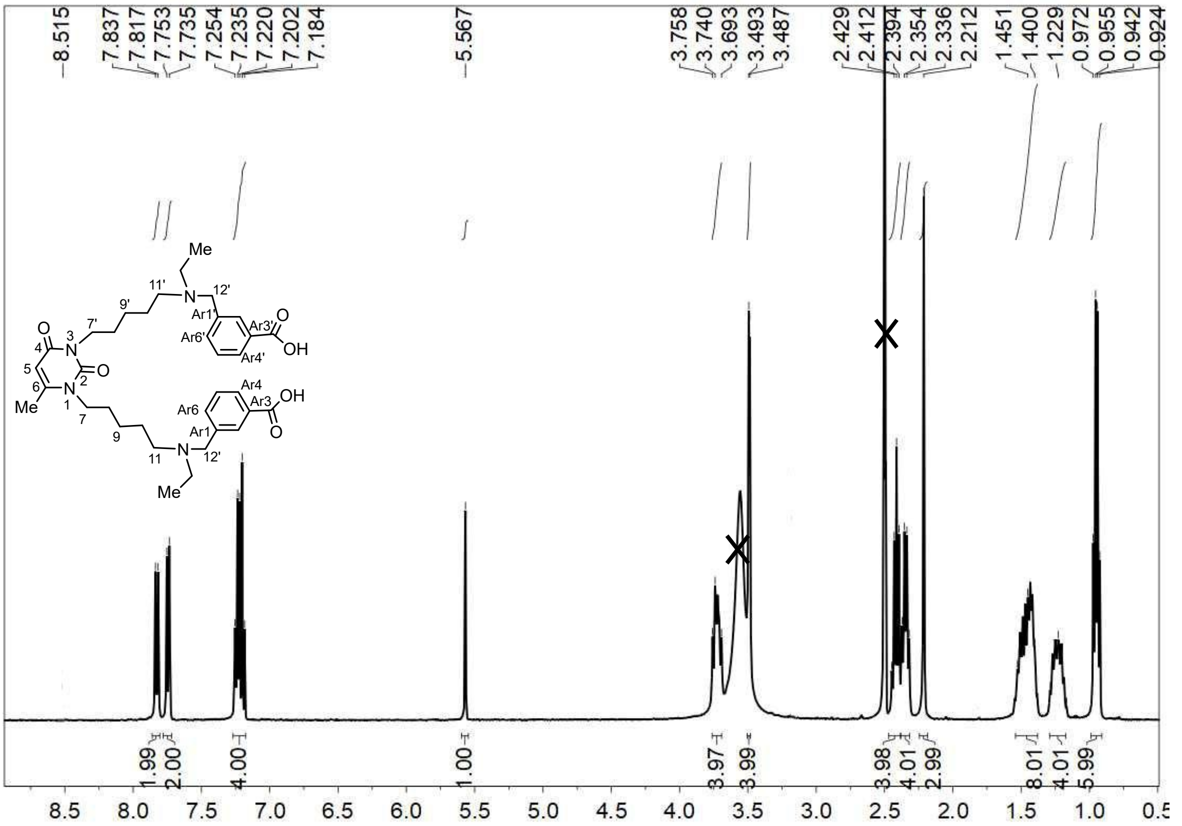

Figure S42. 1D ^{1}H NMR spectrum of **2f** in DMSO-d_6 (400 MHz) at T = 303 K. x - residual solvent peak.

1,3-Bis[5-(m-carboxylbenzylethylamino)pentyl]-6-methyluracil (***2f***): ^{1}H NMR (DMSO-d_6, 400 MHz) δ 7.84, 7.82 (both s, 1H each, 2H, Ar2H, Ar2'H), 7.76-7.73 (m, 2H, Ar5H, Ar5'H), 7.25-7.18 (m, 4H, Ar4H, Ar4'H, Ar6H, Ar6'H), 5.57 (s, 1H, C5H), 3.76-3.69 (m, 4H, N3C7'H_2, N1C7H_2), 3.49, 3.48 (both s, 2H each, 4H, C12CH_2, C12'CH_2), 2.45-2.39, 2.37-2.31 (both m, 4H each, 8H, C11CH_2, C11'CH_2, 2NC$\underline{H_2}$$CH_3$), 2.21 (s, 3H, C6$CH_3$), 1.53-1.40 (m, 8H, C8$CH_2$, C10$CH_2$, C8'$CH_2$, C10'$CH_2$), 1.29-1.17 (m, 4H, C9$CH_2$, C9'$CH_2$), 0.97-0.92 (m, 6H, 2N$CH_2C\underline{H_3}$).
The figure S43 shows ^{1}H NMR spectrum of control, residue from sodium phosphate buffer without AChE in $CDCl_3$. The resonance signals on this spectrum correspond to the signals in the spectrum of compound **2c** (Figure S41). The treatment of compound **2c** with AChE did not cause any changes in the ^{1}H NMR spectrum of the residue (Figure S44). It is also almost identical to the spectrum of pure compound **2c**. Both in Figure S43 and Figure S44 there are no signals of acid forms that are obtained as a result of hydrolysis of ester groups (Figure S42).

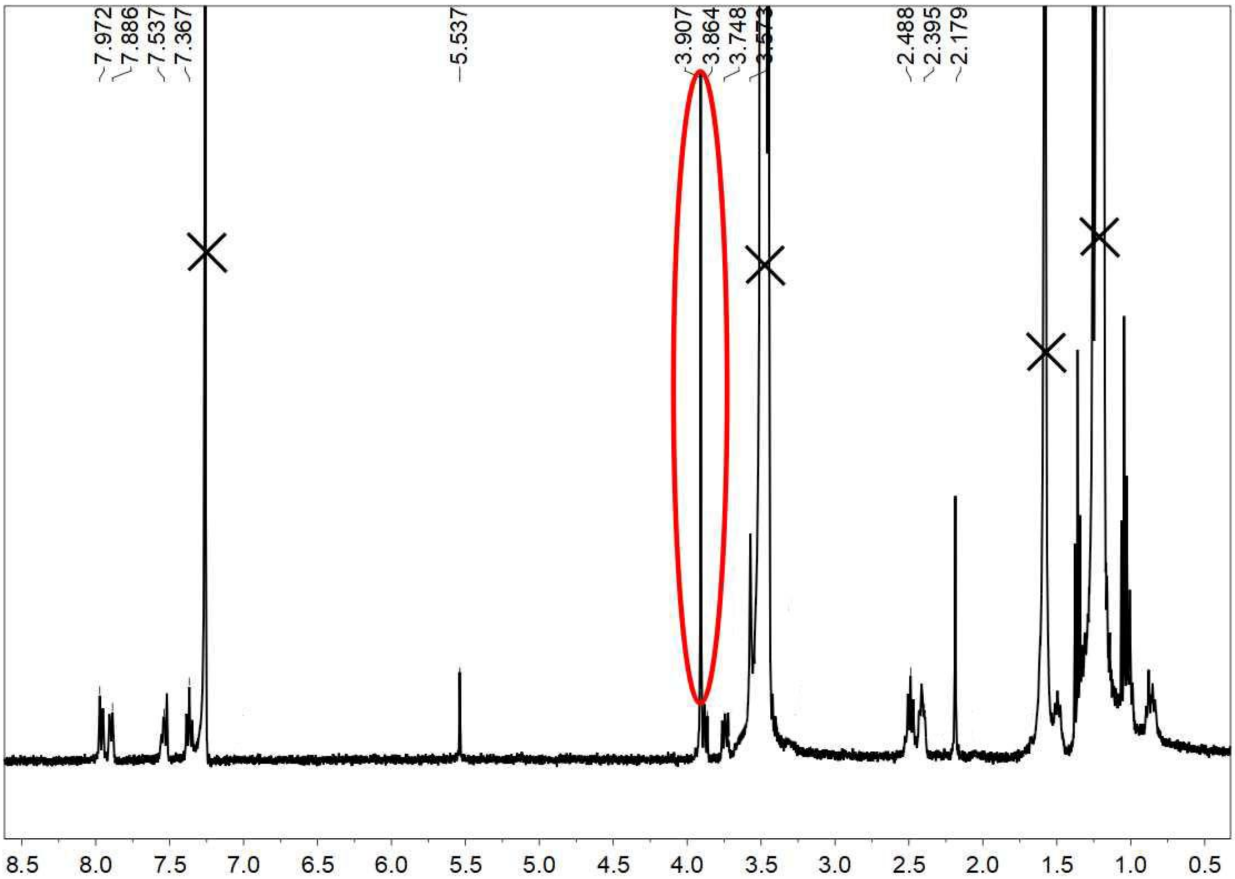

Figure S43. 1D ^{1}H NMR spectrum of residue from sodium phosphate buffer in $CDCl_3$ (600 MHz) at T = 303 K. x - residual solvent peaks. The signal of methyl radicals in ester groups is highlighted with a red ellipse.

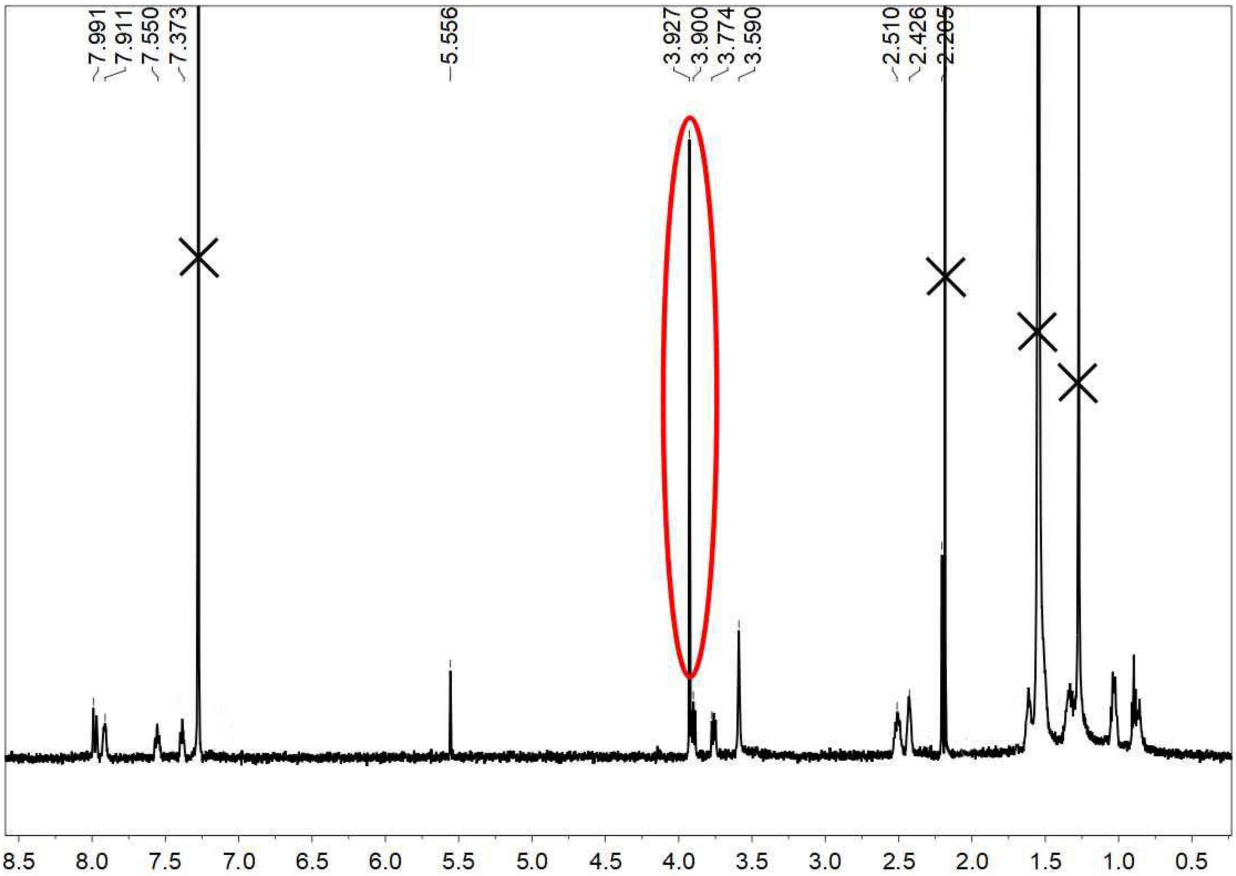

Figure S44. 1D ^{1}H NMR spectrum of residue from sodium phosphate buffer after AChE treatment in $CDCl_3$ (600 MHz) at T = 303 K. x - residual solvent peaks. The signal of methyl radicals in ester groups is highlighted with a red ellipse.
